# Effects of Low pH on Photosynthesis, Related Physiological Parameters, and Nutrient Profiles of *Citrus*

**DOI:** 10.3389/fpls.2017.00185

**Published:** 2017-02-21

**Authors:** An Long, Jiang Zhang, Lin-Tong Yang, Xin Ye, Ning-Wei Lai, Ling-Ling Tan, Dan Lin, Li-Song Chen

**Affiliations:** ^1^Institute of Plant Nutritional Physiology and Molecular Biology, College of Resources and Environment, Fujian Agriculture and Forestry UniversityFuzhou, China; ^2^Fujian Provincial Key Laboratory of Soil Environmental Health and Regulation, College of Resources and Environment, Fujian Agriculture and Forestry UniversityFuzhou, China; ^3^The Higher Educational Key Laboratory of Fujian Province for Soil Ecosystem Health and Regulation, Fujian Agriculture and Forestry UniversityFuzhou, China

**Keywords:** chlorophyll a fluorescence, *Citrus grandis*, *Citrus sinensis*, low pH, OJIP transient, photosynthesis, uptake of nutrient and water

## Abstract

Seedlings of “Xuegan” (*Citrus sinensis*) and “Sour pummelo” (*Citrus grandis*) were irrigated daily with a nutrient solution at a pH of 2.5, 3, 4, 5, or 6 for 9 months. Thereafter, the following responses were investigated: seedling growth; root, stem, and leaf concentrations of nutrient elements; leaf gas exchange, pigment concentration, ribulose-1,5-bisphosphate carboxylase/oxygenase activity and chlorophyll a fluorescence; relative water content, total soluble protein level, H_2_O_2_ production and electrolyte leakage in roots and leaves. This was done (*a*) to determine how low pH affects photosynthesis, related physiological parameters, and mineral nutrient profiles; and (*b*) to understand the mechanisms by which low pH may cause a decrease in leaf CO_2_ assimilation. The pH 2.5 greatly inhibited seedling growth, and many physiological parameters were altered only at pH 2.5; pH 3 slightly inhibited seedling growth; pH 4 had almost no influence on seedling growth; and seedling growth and many physiological parameters reached their maximum at pH 5. No seedlings died at any given pH. These results demonstrate that citrus survival is insensitive to low pH. H^+^-toxicity may directly damage citrus roots, thus affecting the uptake of mineral nutrients and water. H^+^-toxicity and a decreased uptake of nutrients (i.e., nitrogen, phosphorus, potassium, calcium, and magnesium) and water were likely responsible for the low pH-induced inhibition of growth. Leaf CO_2_ assimilation was inhibited only at pH 2.5. The combinations of an impaired photosynthetic electron transport chain, increased production of reactive oxygen species, and decreased uptake of nutrients and water might account for the pH 2.5-induced decrease in CO_2_ assimilation. Mottled bleached leaves only occurred in the pH 2.5-treated *C. grandis* seedlings. Furthermore, the pH 2.5-induced alterations of leaf CO_2_ assimilation, water-use efficiency, chlorophylls, polyphasic chlorophyll a fluorescence (OJIP) transients and many fluorescence parameters, root and leaf total soluble proteins, H_2_O_2_ production, and electrolyte leakage were all slightly greater in *C. grandis* than in *C. sinensis* seedlings. Hence, *C. sinensis* was slightly more tolerant to low pH than *C. grandis*. In conclusion, our findings provide novel insight into the causes of low pH-induced inhibition of seedling growth and leaf CO_2_ assimilation.

## Introduction

Acidic soils that limit crop growth and productivity are often observed all over the world, especially in the tropics and subtropics. Approximately 30% of the world's ice-free land is acidic, and approximately 12% of crops are cultivated on acidic soils (von Uexküll and Mutert, 1995). What is worse, soil acidification is becoming an increasingly major problem due to the improper application of chemical fertilizers—particularly the overuse of nitrogen (N) fertilizers—alongside acid rain and intensive agriculture and monoculture (Wu et al., 2013; Yang et al., 2013). The effects of aluminum (Al)-toxicity—a major factor limiting crop productivity on acidic soils—on plants have drawn widespread attention, but few studies have investigated the damage to plants from low pH (Yang et al., 2015).

Poor crop growth and yield on acidic soils is usually due to the combination of toxicities of H^+^, Al, and manganese (Mn) and a lack of nutrients—namely phosphorus (P), calcium (Ca), magnesium (Mg), potassium (K), and molybdenum (Mo)— and a reduced uptake of water (von Uexküll and Mutert, 1995; Bian et al., 2013). In tropical America, over 70% of the acidic soils display Al-toxicity and Mg and Ca deficiencies, and almost all the acidic soils are P-deficient or have a high P-fixation capacity (George et al., 2012). For example, Zhang et al. (2014) showed that pH 3.0 decreased the uptake and utilization efficiency of P in *Juglans regia* seedlings. Forest ecosystems with acidic soils are often restricted by low Ca and Mg availability (St Clair and Lynch, 2005). Schubert et al. (1990) showed that transferring *Vicia faba* plants from pH 7 to pH 4 led to the reduced uptake of N, P, K, Ca, Mg, and sulfur (S). Malkanthi et al. (1995) observed that the levels of K, Ca, Mg, Mn, and Zn in the roots and tops of wheat, barley, and chili plants were lower at pH 3.8 than at pH 5.5. Similarly, the K, Ca, Mg, and Mn levels in *Pinus pinaster* roots and needles were lower at pH 3.5 than at pH 4.5, 5.5, and 6.5, whereas the levels of P and Fe were higher at pH 3.5 and 4.5 than at pH 5.5 and 6.5 (Arduini et al., 1998). However, Anugoolprasert et al. (2012) reported that the uptake of N, P, K, Ca, and Mg, and their concentration in roots, leaflets, petioles and whole plant, were not altered over the range of pH 3.6 to 5.7 for 4.5 months; this possibly explains the normal growth of sago palm seedlings at pH 3.6. Kidd and Proctor (2001) have suggested that the direct toxicity of H^+^ was the primary cause of the poor growth in H^+^-intolerant plants growing in very acidic soils.

Low pH can affects plant water uptake. Kamaluddin and Zwiazek (2004) observed that low pH caused a large and rapid decrease in both the water flow rate and the hydraulic conductivity in seedling roots of paper birch (*Betula papyrifera*). A pH 4.5 decreased the whole-root water conductivity in the H^+^-sensitive maize cultivar *Adour 250*, but it did not in the H^+^-tolerant maize cultivar *BR 201 F* (Gunsé et al., 1997). Tournaire-Roux et al. (2003) showed that the inhibition of water hydraulic conductivity (water uptake) in *Arabidopsis* roots by anoxia was primarily caused by cytosol acidosis, while changing the pH between 5.5 and 8.0 of a root-bathing solution did not affect the cytosol pH nor the root water hydraulic conductivity. Finally, Yang M. et al. (2011) observed that a low pH decreased the water content in *Eucalyptus* roots, stems, and leaves.

Low pH also inhibits CO_2_ assimilation in some plant species, including *J. regia* (Zhang et al., 2014), *Eucalyptus* (Yang et al., 2015), sugar maple (*Acer saccharum*) and red maple (*Acer rubrum*) (Ellsworth and Liu, 1994; St Clair and Lynch, 2005). St Clair and Lynch (2005) also reported that the base cation stimulation of photosynthesis in sugar maple on acidic soils was correlated with its foliar nutrient status. Ellsworth and Liu (1994) had earlier suggested that photosynthesis in sugar maple on acidic soils might be co-limited by N and Ca, or by Ca × Mg interactions. Yang M. et al. (2011) observed that a low pH decreased the chlorophyll (Chl) level in *Eucalyptus* leaves. Yang et al. (2015) further investigated the effects of low pH on leaf gas exchange and Chl in four vegetatively-propagated *Eucalyptus* clones (G9, G12, G3, and G4); they found that pH 3.0 decreased leaf photosynthesis, transpiration, and Chl level in the four clones as well as the leaf water-use efficiency (WUE) in the G4 leaves, but pH 3.0 did not affect WUE in the G9, G12, and G3 leaves. Zhang et al. (2014) reported that pH 3.0 decreased the leaf net photosynthetic rate, transpiration rate, actual quantum yield of the photosystem II (PSII) electron transport (Φ_PSII_), whereas it increased leaf non-photochemical quenching (NPQ); however, pH 3 had no effect upon leaf stomatal conductance, photochemical quenching (qP), and the maximum PSII efficiency of dark-adapted leaves (F_v_/F_m_), thus leading the authors to conclude that non-stomatal factors played a role in the low pH-induced inhibition of photosynthesis. Nonetheless, pH 4.0 did not influence spatial heterogeneity of Chl fluorescence, F_v_/F_m_, Φ_PSII_, and quantum yields of regulated (Φ_NPQ_) and nonregulated (Φ_NO_) energy dissipation in the leaves of *Plantago algarbiensis* and *P. almogravensis* (Martins et al., 2013a,c). Altering the pH between 5.7 and 3.6 did not reduce the Chl concentration, photosynthetic rate, stomatal conductance, and transpiration rate in sago palm leaves (Anugoolprasert et al., 2012). However, to our best knowledge, little is still known about the effects of low pH on PSII photochemistry (i.e., absorption flux, trapped energy flux, electron flux, and dissipated energy flux) of leaves.

Low pH can induce oxidative stress and electrolyte leakage via the enhanced production of active oxygen species (ROS). Martins et al. (2013b) found that lipid peroxidation (malondialdehyde, MDA) was elevated in the pH 4.0-treated *P. algarbiensis* shoots, but not in the pH 4.0-treated *P. almogravensis* ones, and that the activities of antioxidant enzymes were enhanced or not affected in the shoots of the two *Plantago* species—suggesting that the higher antioxidant enzyme activities were insufficient to protect the low pH-treated *P. algarbiensis* shoots against oxidative damage. In another experiment, Martins et al. (2011) observed that pH 4.5 led to an increase in the MDA level in *P. algarbiensis* roots and shoots and *P. almogravensis* roots, but not in *P. almogravensis* shoots. Yang M. et al. (2011) reported that low pH increased membrane permeability in *Eucalyptus* leaves. Hydroponic experimentation showed that pH 3.5 led to an accumulation of H_2_O_2_ and severe lipid peroxidation that was accompanied by an increased activity of ascorbate peroxidase (APX) and decreased activities of superoxide dismutase (SOD) and catalase (CAT) in the roots of two rice cultivars (Zhang et al., 2015). Cucumber roots treated with pH 4.5 had a higher level of MDA and activities of monodehydroascorbate reductase (DHAR), guaiacol peroxidase (GPX), APX, and glutathione reductase (GR), but had lower activities of Cu/Zn-SOD, than did the pH 6.5-treated roots (Shi et al., 2006). However, pH 4.0 did not affect H_2_O_2_, MDA and the total soluble protein levels, electrolyte leakage, protein oxidation, and the SOD, CAT, APX, and GPX activities in the roots and leaves of *P. algarbiensis* and *P. almogravensis* (Martins et al., 2013c).

Citrus plants are considered insensitive to acidic soils (Yuda and Okamoto, 1965). Fang et al. (2011) used a solution culture approach to investigate the effects of pH 1.0, 2.0, 3.0, 4.0, 5.0, and 6.0 on several citrus rootstock seedlings. At pH 1.0, all seedlings died within 10 days after treatment, but the pH 4-treated seedlings showed normal growth except for a yellow tip that occurred in some leaves within 30 days. Using sand and solution cultures, Guest and Chapman (1944) found that *Citrus sinensis* seedlings died within a few days at pH 2.0, but they were not killed for months at pH 2.5 and 3.0 though their growth was limited or negligible. Nevertheless, citrus do not thrive in trongly acidic soils, because serious problems may arise when the soil pH is 5.0 or lower (Chapman, 1968). Citrus will often display poor growth and have a shortened lifespan when cultivated on soil with a low pH and high active Al (Lin and Myhre, 1990). In China, most of the citrus are grown in acidic and strongly acidic soils. Li et al. (2015) reported that the pH values of 319 soils sampled from pummelo (*Citrus grandis*) orchards in Pinghe, Zhangzhou, China had an average value of 4.34 and ranged from 3.26 to 6.22, with up to 90.0% of the orchard soils having a pH lower than 5.0. So far, however, only a handful of reports have empirically investigated the effects of low pH on citrus growth (Yuda and Okamoto, 1965), mineral nutrient uptake (Randhawa and Iwata, 1968; He et al., 1999; Li et al., 2015), and ROS metabolism alongside a few other physiological parameters (Fang, 2011). Randhawa and Iwata (1968) reported that the N, Ca, and Mg (Ca, Mg, and P) levels decreased in the leaves (roots), whereas the K level increased in the roots and leaves of *Citrus natsudaidai* seedlings, as the pH decreased from 7.0 to 4.0. He et al. (1999) observed that Fe, Zn, and Mn (Ca) in grapefruit (*Citrus paradisi*) leaves increased (decreased) with decreasing soil pH. The concentration of P and Ca in pummelo leaves decreased with decreasing soil pH (Li et al., 2015). Fang (2011) found that the activities of SOD, GPX, and CAT and the level of total soluble proteins displayed an upward trend, as a whole, as the pH decreased from 6.0 to 2.0; in contrast, the level of MDA decreased first to reach its lowest value at pH 4, but then increased as the pH decreased further.

The objectives of this work were (*a*) to determine how low pH affects gas exchange, related physiological parameters, and the mineral nutrient profiles in citrus seedlings; and (*b*) to understand the mechanisms by which low pH may lead to a decrease in leaf CO_2_ assimilation.

## Materials and methods

### Plant materials and culture conditions

This study was conducted at the Fujian Agriculture and Forestry University (FAFU) in Fuzhou, China. Seedling culture was performed according to Han et al. (2008) and Peng et al. (2015), with some modifications. Briefly, seeds of “Sour pummelo” (*C. grandis*) and “Xuegan” (*C. sinensis*) were germinated in plastic trays filled with clean river sand. Four weeks after germination, uniform seedlings that had a single stem were chosen and transplanted into 6-L terracotta pots (two seedlings per pot) containing clean river sand. Seedlings were grown in a greenhouse under a natural photoperiod at FAFU. One week after transporting, each pot was irrigated every other day with 500 mL of a nutrient solution containing 2.5 mM Ca(NO_3_)_2_, 2.5 mM KNO_3_, 1 mM MgSO_4_, 0.5 mM KH_2_PO_4_, 20 μM Fe-EDTA, 10 μM H_3_BO_3_, 2 μM MnCl_2_, 2 μM ZnSO_4_, 0.5 μM CuSO_4_, and 0.065 μM (NH_4_)_6_Mo_7_O_24_. Seven weeks after transplanting, each pot was fertilized daily until saturated with the same nutrient solution (approximately 500 mL), except that the pH of the nutrient solution was adjusted to 2.5, 3, 4, 5, or 6 with 1 M HCl. There were 20 replicates (20 pots, 40 seedlings) per treatment in a completely randomized design. In this experiment, the pH 5 treatment served as the control because seedling growth and many physiological parameters reach their maximum at pH 5. Nine months after the pH treatment began, recent fully-expanded (approximately 7-week-old) leaves and approximately 5-mm-long white root apices were used for all measurements except that for root mineral element concentrations. After leaf gas exchange and Chl a fluorescence were measured, leaf disks (0.2826 cm^2^ in size) and approximately 5-mm-long white root apices from the same seedlings were harvested from randomly selected seedling at noon on a sunny day and immediately frozen in liquid N_2_, then stored at −80°C until they were used for the assays of ribulose-1,5-bisphosphate carbohylase/oxygenase (Rubisco), total soluble proteins, and pigments. The remaining seedlings that were not sampled were selected randomly to measure plant biomass, root and leaf relative water content (RWC), and electrolyte leakage, and the root, stem and leaf mineral element concentrations.

### Measurements of leaf, stem and root dry weight (DW), and specific leaf weight

Nine months after the pH treatment began, 10 seedlings per treatment from 10 pots were collected. The seedlings were divided into leaves, stems, and roots. Their DW was measured after being dried at 70°C for 48 h. Specific leaf weight was calculated as the ratio of leaf weight to leaf area.

### Leaf pigments, and root and leaf total soluble proteins

Leaf pigments were extracted with 80% (v/v) acetone. The Chl, Chl a and Chl b, and carotenoids (Car) in the extract were determined according to Lichtenthaler (1987).

Root and leaf total soluble proteins were extracted with 50 mM KH_2_PO_4_-Na_2_HPO_4_ (pH 7.0) and 5% (v/v) insoluble polyvinylpyrrilodone (PVP), and assayed according to Bradford (1976).

### Electrolyte leakage, RWC, and H_2_O_2_ production

Root and leaf electrolyte leakage was assayed according to Han et al. (2008). Briefly, 20 fresh leaf disks (0.2826 cm^2^ in size) from the same leaf or 20 approximately 5-mm-long white root apices taken at midday under full sun, were immediately transferred to a 50-mL tube filled with 15 mL of distilled water. The tubes were placed at room temperature in the dark for 24 h and the first electrical conductance (C_1_) was measured. Then the tubes were incubated in a boiling water bath for 15 min and the second electrical conductance (C_2_) was measured after being cooled. The electrolyte leakage was calculated as: electrolyte leakage (%) = (C_1_/C_2_) × 100.

Root and leaf RWC were gravimetrically determined (Panković et al., 1999). After fresh weight (FW) was measured, approximately 0.2 g of roots and 0.5 g of leaves were floated on distilled water in Petri dishes in the dark. After reaching a constant turgid weight (ca. 6 h), the roots and leaves were dried. The RWC was calculated as: RWC (%) = (FW − DW)/(turgid weight − DW) × 100.

Root and leaf H_2_O_2_ production were determined according to Chen et al. (2005b). About 100 mg of roots and 15 leaf disks (0.2826 cm^2^ in size) were incubated in 2 mL of a 50 mM phosphate buffer (pH 7.0), 5 U horseradish GPX, and 0.05% (w/v) guaiacol for 2 h at room temperature in the dark. Then the absorbance was measured at 470 nm.

### Measurements of mineral elements, and the calculation of nutrient uptake and element distribution in roots, stems, and leaves

Fibrous roots, the middle sections of stems, and approximately 7-week-old leaves (midribs and petioles removed) were collected and dried at 70°C for 48 h. Dried samples were ground in a mortar to pass through a 40-mesh sieve and stored for later analysis.

To measure the root, stem, and leaf concentrations of P, K, Fe, Mn, Cu, Zn, Ca, and Mg, approximately 0.3-g samples were digested in a 7 mL mixture of HNO_3_:H_2_O_2_ (5:2 v/v). P was determined colorimetrically as the blue molybdate-phosphate complexes according to Lu (1999). K was assayed using FP640 Flame Photometry (Shanghai Precision Scientific Instrument Co., Ltd, Shanghai, China). Fe, Mn, Cu, Zn, Ca, and Mg were determined using a PinAAcle 900F Atomic Absorption Spectrometer (Perkinelmer Singapore Pte Ltd, Singapore). N was measured using a Kjeltec 8200 Auto Distillation (FOSS Analytical AB, Höganäs, Sweden) after samples had been digested with H_2_SO_4_ and H_2_O_2_ (Lu, 1999). B was determined by the curcumin method after samples were ashed at 500°C for 5 h and dissolved in 0.1 M HCl (Kowalenko and Lavkulich, 1976). S was assayed using the simple turbidimetric method based on the formation of the BaSO_4_ precipitate in its colloid form after approximately 0.3-g samples were digested with a 6-mL mixture of HNO_3_:HClO_4_ (4:1 v/v; Lu, 1999).

Nutrient uptake per plant was the sum of the element content (element concentration × tissue DW) in the roots, stems, and leaves. Element distributions in roots, stems, or leaves (%) were calculated as: (element content in roots, stems, or leaves/the sum of element content in roots, stems, and leaves) × 100.

### Leaf gas exchange and rubisco measurements

Leaf gas exchange was measured by a CIARS-2 portable photosynthesis system (PP Systems, Herts, UK) at an ambient CO_2_ concentration under a controlled light intensity of 996–1004 μmol m^−2^ s^−1^ between 9:30 and 12:30 on a clear day. During all of these measurements, the leaf temperature and relative humidity were 30.0 ± 0.2°C and 64.5 ± 0.6%, respectively. Leaf Rubisco was extracted and assayed according to Chen et al. (2005a) and Lin et al. (2009), respectively.

### Measurements of leaf OJIP transients by handy PEA and the JIP test

The polyphasic Chl a fluorescence (OJIP) transients were measured by a Handy Plant Efficiency Analyzer (Handy PEA, Hansatech Instruments Limited, Norfolk, UK). The transient was induced by a saturating red light of approximately 3,400 μmol m^−2^ s^−1^, which was provided by an array of three light-emitting diodes (peak 650 nm) that were focused on the leaf surface to provide homogenous illumination over the exposed area of the leaf. All the measurements were performed on 3-h dark-adapted plants at room temperature.

The OJIP transients were analyzed according to the JIP test (Strasser et al., 2004; Jiang et al., 2008; Chen and Cheng, 2009). The following data from the original measurements were extracted and used: fluorescence intensities at 20 μs (F_20μ*s*_, considered as the minimum fluorescence F_o_), 50 μs (F_50μ*s*_), 300 μs (F_300μ*s*_), 2 ms (J-step, F_J_), 30 ms (I-step, F_I_), and P-step (considered as the maximum fluorescence F_m_). The following parameters that refer to “time 0” (start of fluorescence induction) are: (*a*) fluorescence parameters derived from the extracted data, i.e., the maximum variable fluorescence F_v_ = F_m_ − F_o_ and the approximated initial slope (in ms^−1^) of the fluorescence transient V = f(t) [M_o_ = 4(F_300μ*s*_−F_o_)/(F_m_−F_o_)]; (*b*) the specific energy fluxes per reaction center (RC) for energy dissipation (DI_o_/RC) and absorption (ABS/RC); (*c*) the yields of the flux ratios, i.e., quantum yield for energy dissipation (φ_Do_ = DI_o_/ABS = F_o_/F_m_), maximum quantum yield of primary photochemistry (φ_Po_ = TR_o_/ABS = F_v_/F_m_), quantum yield for the reduction of the end acceptors of photosystem I (PSI) per photon absorbed (φ_Ro_ = RE_o_/ABS), and quantum yield for electron transport (φ_Eo_ = ET_o_/ABS); (*d*) the overall grouping probability (P_2G_); and (*e*) the total performance index (PI_tot, abs_).

### Measurements of conventional fluorescence parameters by FMS-2

Conventional fluorescence parameters were determined by a pulse-modulated fluorometer FMS-2 (Hansatech Instruments, Norfolk, UK). Both F_m_ and F_o_ were measured after the leaves were dark-adapted for 40 min. Steady-state fluorescence (F_s_) and the maximum (F_m_′) and minimum (F_o_′) fluorescences were measured under natural light at midday in full sun. For this determination, the F_s_ was monitored to ensure it was stable before a reading was taken; the F_m_′ was obtained by imposing a 1-s saturating flash of approximately 6,000 μmol m^−2^ s^−1^ at the leaf surface to reduce all the PSII centers. To measure the F_o_′, a black cloth covered the leaf when a far-red light was switched on to rapidly oxidize the PSII centers by drawing electrons from PSII to PSI. The NPQ was calculated as: F_m_/F_m_′−1. The photochemical quenching coefficient, qP, was expressed as: (F_m_′−F_s_)/(F_m_′−F_o_′). The non-photochemical quenching coefficient, qNP, was defined as: (F_m_−F_m_′)/(F_m_−F_o_′). The Φ_PSII_ was calculated as: (F_m_′−F_s_)/F_m_′. The efficiency of excitation transfer to PSII RCs under natural light (F_m_′/F_v_′) was defined as: (F_m_′−F_o_′)/F_m_′. Finally, the electron transport rate through PSII was estimated from (F_m_′−F_s_)/F_m_′ × 0.5 × LA × photosynthetic photon flux (PPF), for which the PSI photochemistry was assumed equivalent to that of PSII (Genty et al., 1990), and where LA is the leaf absorbance (0.84; Baker, 2008).

### Statistical analysis

There were 10 replicates for plant biomass; three replicates for Rubisco; four replicates for gas exchange, pigments, H_2_O_2_ production, RWC, electrolyte leakage, total soluble proteins, specific leaf weight, and mineral nutrients; and 7–15 replicates for the OJIP transients and the fluorescence parameters. The results are presented using the mean ± SE of 3–15 replicates. For a given dependent variable or parameter above, significant differences among the means of 10 treatment combinations were tested by a two (species) × five (pH levels) factorial ANOVA; the 10 means were compared on a pairwise basis by the Duncan's new multiple range test at *P* < 0.05. Linear and nonlinear regression was performed with the corresponding equations from SigmaPlot software (SigmaPlot 10.0, Systat Software Inc., USA).

## Results

### Effects of pH on seedling growth

**Figure 1:**
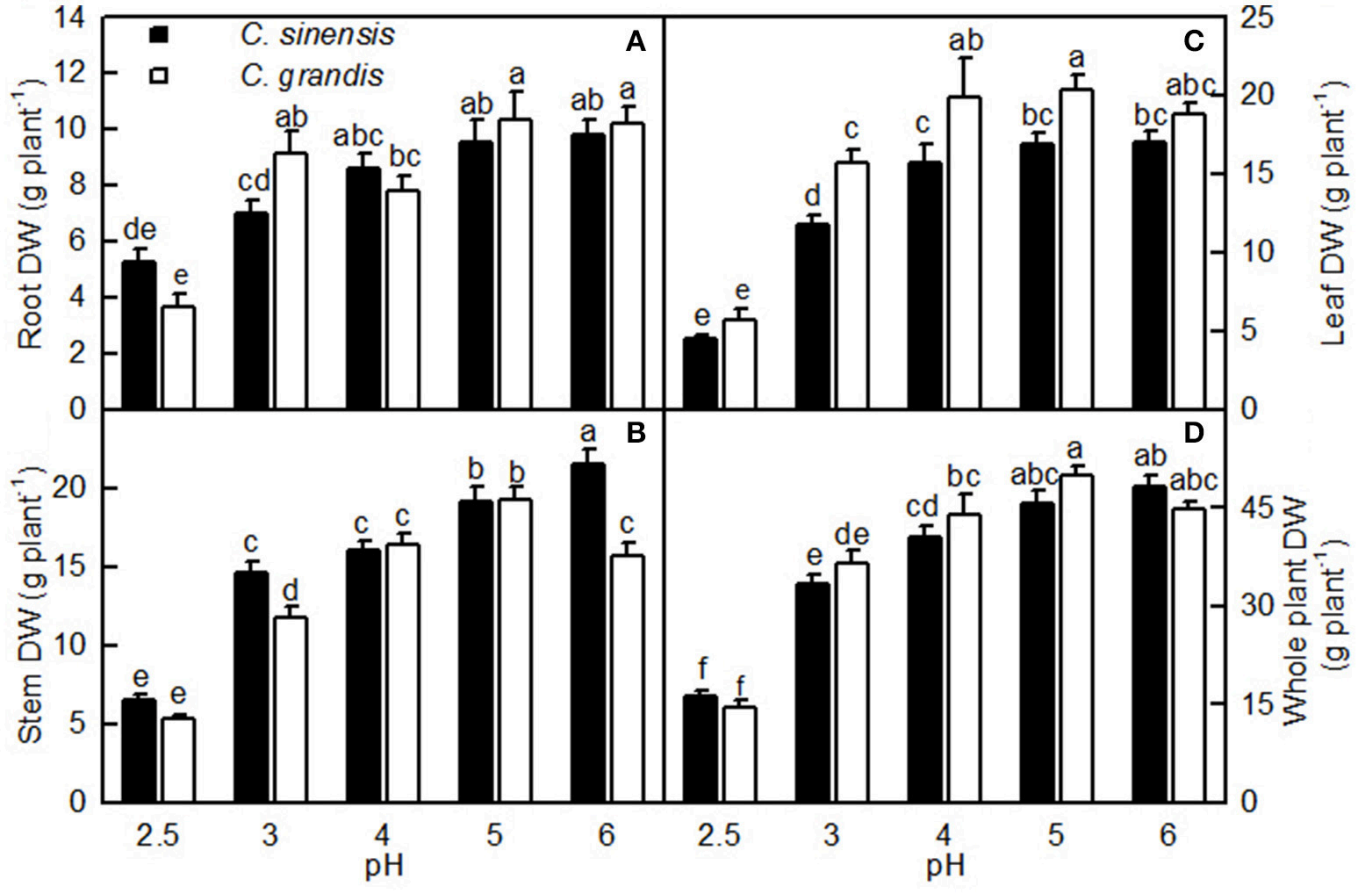
**Effects of pH on root (A), stem (B), leaf (C), and whole plant (D) DW of *Citrus sinensis* and *Citrus grandis* seedlings**. Bars represent means ± SE (*n* = 10). Differences among the 10 treatments were analyzed by two (species) × five (pH) factorial ANOVA. Different letters above the bars indicate a significant difference at *P* < 0.05.

**Figure 2:**
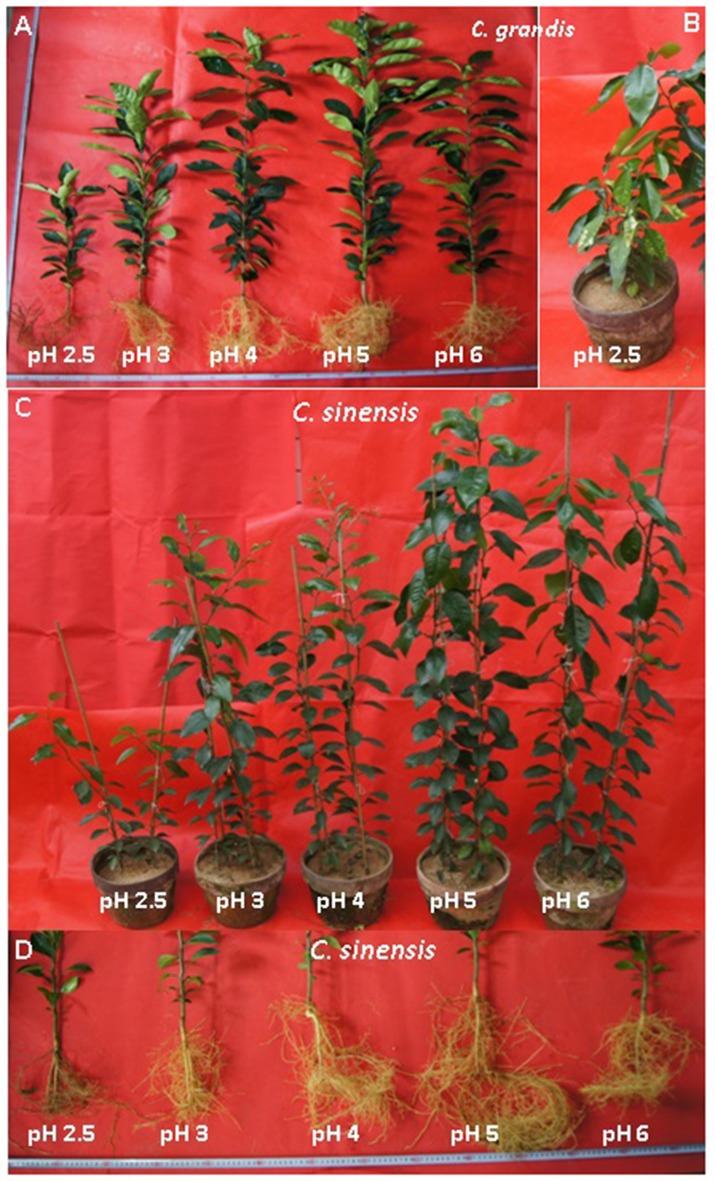
**Effects of pH on the growth of *Citrus grandis* (A,B) and *Citrus sinensis* (C,D) seedlings**.

Overall, the pH-2.5 treatment greatly decreased root, stem, leaf, and whole plant DW; pH 3 slightly inhibited seedling growth; pH 4 had almost no influence on seedling growth; and seedling growth reached a maximum at pH 5 (Figures 1, 2). At pH 2.5, many rotted fibrous roots were observed, and the living roots had turned abnormally dark brown (Figures 2A,D). Mottled bleached leaves were found in four *C. grandis* seedlings treated with pH 2.5 (Figure 2B). No seedling death was observed for the two citrus species at each given pH.

### Effects of pH on leaf gas exchange, rubisco activity, and pigment levels

**Figure 3:**
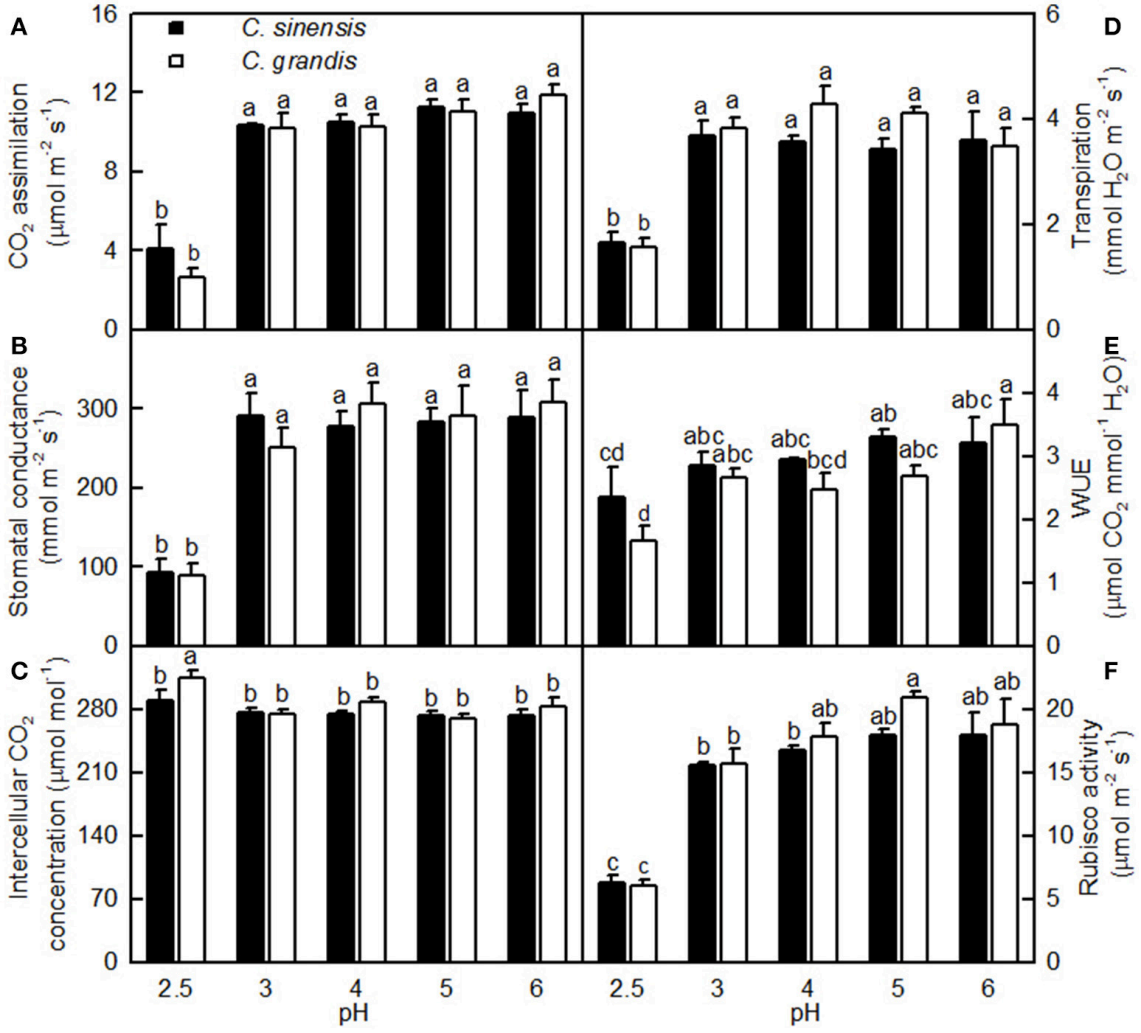
**Effects of pH on CO_2_ assimilation (A), stomatal conductance (B), intercellular CO_2_ concentration (C), transpiration rate (D), water-use efficiency (WUE, E), and Rubisco activity (F) in *Citrus sinensis* and *Citrus grandis* leaves**. Bars represent means ± SE (*n* = 3 for Rubisco or *n* = 4 for the other parameters). Differences among the 10 treatments were analyzed by two (species) × five (pH) factorial ANOVA. Different letters above the bars indicate a significant difference at *P* < 0.05.

As shown in Figure 3, leaf CO_2_ assimilation, stomatal conductance, transpiration, and Rubisco activity were little changed as the pH decreased from 6 to 3, but they greatly decreased at pH 2.5. Leaf WUE was lower at pH 2.5 than at pH 5. All five parameters were similar between the two citrus species at each given pH. Intercellular CO_2_ concentration did not significantly differ among the 10 treatment combinations, but there was a slight increase observed in the pH 2.5-treated *C. grandis* leaves.

**Figure 4:**
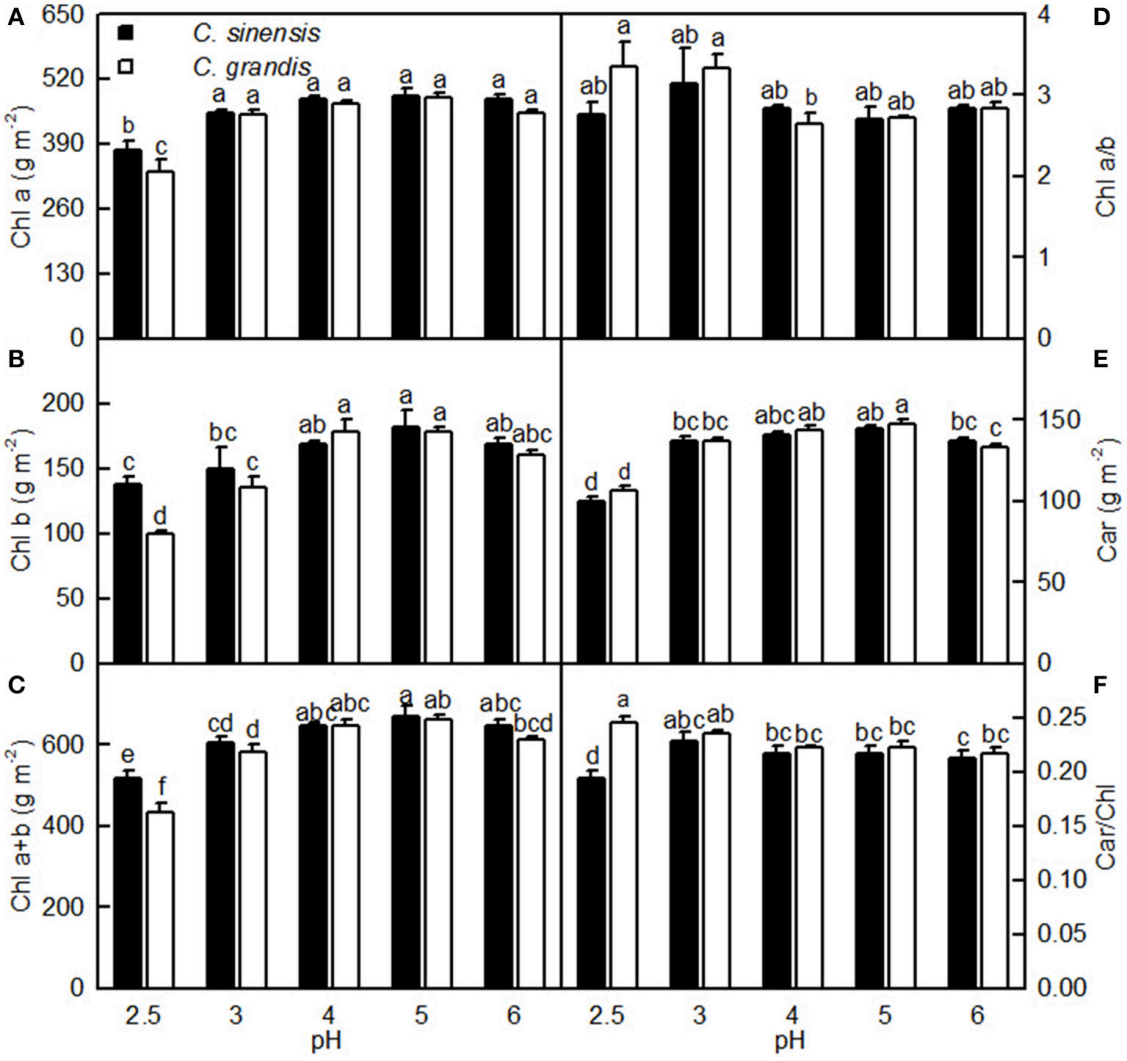
**Effects of pH on Chl a (A), Chl b (B), Chl a+b (C), Chl a/b (D), Car (E), and Car/Chl (F) in *C. sinensis* and *C. grandis* leaves**. Bars represent means ± SE (*n* = 4). Differences among the 10 treatments were analyzed by two (species) × five (pH) factorial ANOVA. Different letters above the bars indicate a significant difference at *P* < 0.05.

As shown in Figure 4, leaf Chl a, Chl b, Chl a+b, and Car concentrations greatly increased as the pH increased from 2.5 to 3, after which they remained unchanged or were only slightly altered with increasing pH. These concentrations did not differ significantly between the two citrus species at pH 3, 4, 5, and 6, but they were lower in *C. sinensis* leaves than in *C. grandis* leaves at pH 2.5. Moreover, there was little difference in the ratios of leaf Chl a/b and Car/Chl among the 10 treatment combinations. The only exception was the lower Car/Chl ratio in the pH 2.5-treated *C. sinensis* leaves when compared with the other nine treatment combinations.

**Figure 5:**
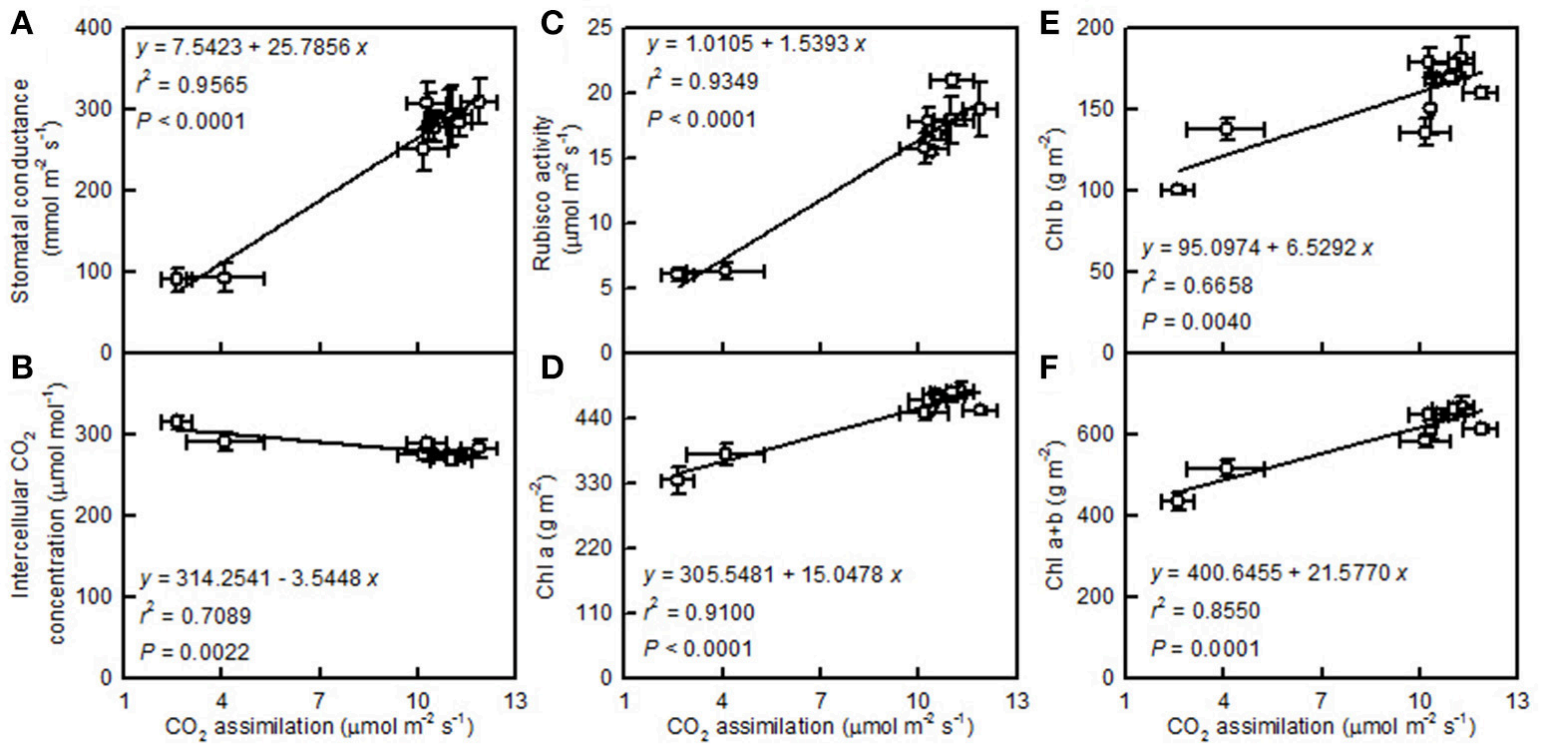
**Leaf CO_2_ assimilation in relation to stomatal conductance (A), intercellular CO_2_ concentration (B), Rubisco activity (C), Chl a (D), Chl b (E), and Chl a+b (F)**. Points represent means ± SE for the independent variable (*n* = 4) and the dependent variables (*n* = 3 or 4). Data for CO_2_ assimilation, stomatal conductance, intercellular CO_2_ concentration, and Rubisco activity are from Figure 3. Data for Chl a, Chl b, and Chl a+b are from Figure 4. Data for the two citrus species were pooled together.

Leaf CO_2_ assimilation increased with increasing leaf stomatal conductance, the activity of Rubisco, and the concentration of Chl a, Chl b, or Chl a+b, but it decreased with an increasing intercellular CO_2_ concentration (Figure 5).

### Effects of pH on Chl a fluorescence and related parameters

**Figure 6:**
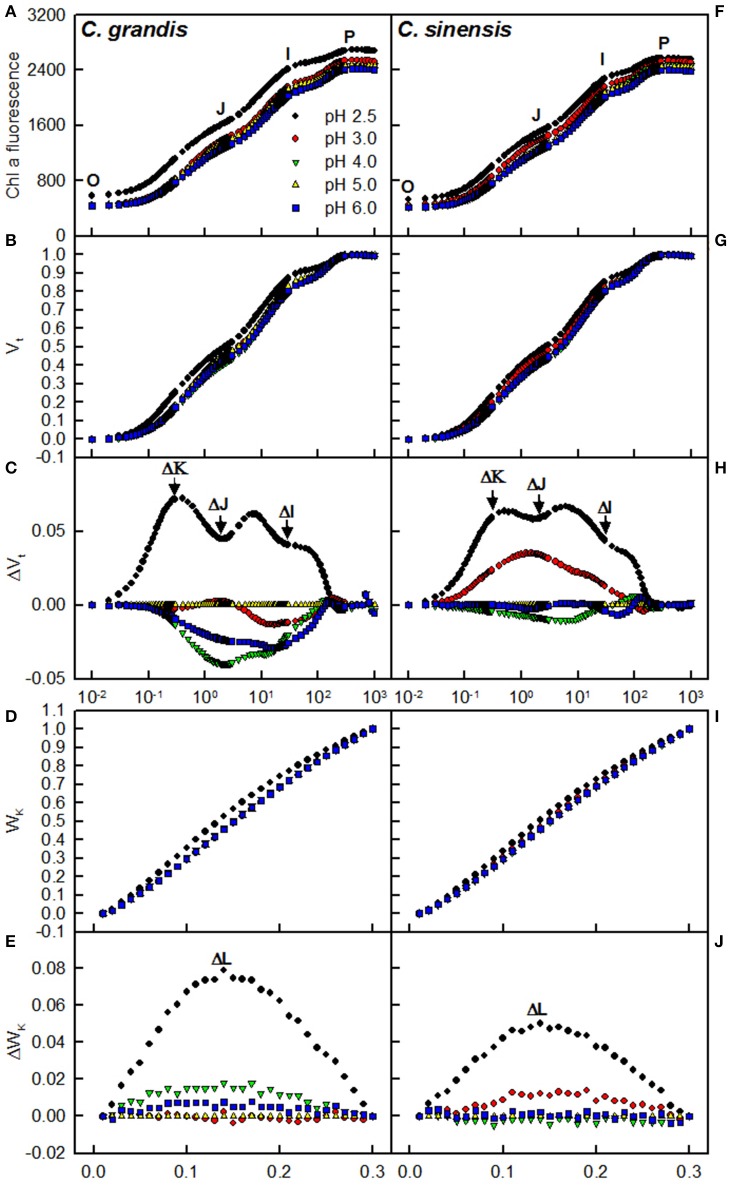
**Effects of pH on the mean chlorophyll a fluorescence (OJIP) transients (A,F) and the different expressions derived from the transients in dark-adapted leaves: (B,G) between F_o_ and F_m_: V_t_ = (F_t_−F_o_)/(F_m_−F_o_) and (C,H) the differences of the five samples to the reference sample treated with pH 5.0 (ΔV_t_); (D,I) between F_o_ and F_300μ*s*_: W_K_ = (F_t_−F_o_)/(F_300μ_−F_o_) and (E,J) the differences of the five samples to the reference sample treated with pH 5.0 (ΔW_K_)**. Each point was the mean of 8–15 replicates.

Our results showed that pH 2.5 caused an increased O-step and P-step in *C. sinensis* and *C. grandis* leaves compared with pH 5, and that the pH 2.5-treated *C. sinensis* and *C. grandis* leaves had positive ΔI-, ΔJ-, ΔK-, and ΔL-bands around 30 ms, 2 ms, 300 μs, and 130 μs as compared with the pH 5-treated leaves, respectively. The pH 2.5-induced alterations of the OJIP transients and the ΔI- and ΔL-bands were greater in the leaves of *C. grandis* than in those of *C. sinensis*. Little, if any, differences were observed in the OJIP transients among the pH 3-, 4-, 5-, and 6-treated leaves (Figure 6).

**Figure 7:**
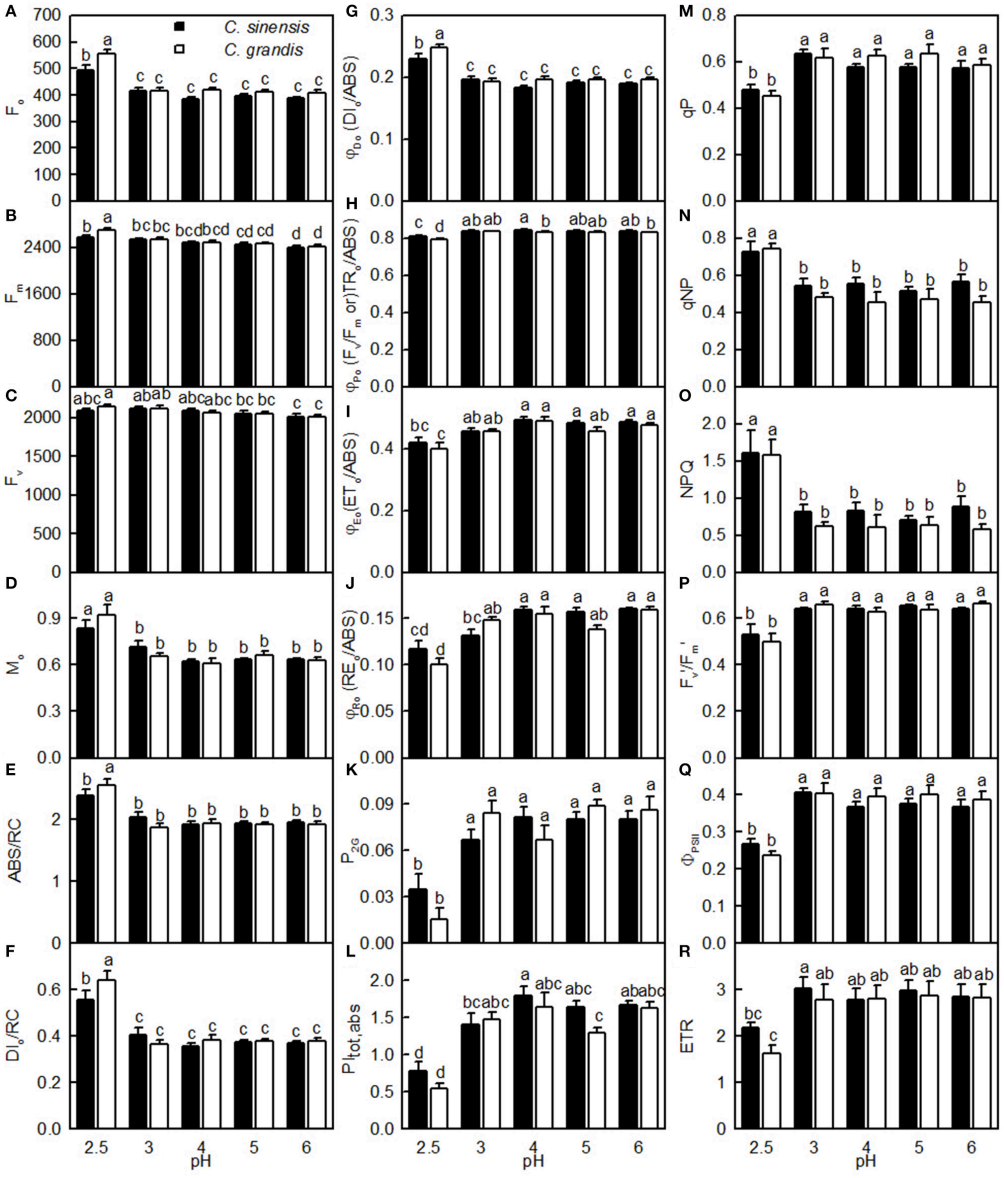
**Effects of pH on F_o_ (A), F_m_ (B), F_v_ (C), M_o_ (D), ABS/RC (E), DI_o_/RC (F), DI_o_/ABS (G), F_v_/F_m_ (H), ET_o_/ABS (I), RE_o_/ABS (J), P_2G_ (K), PI_tot, abs_ (L), qP (M), qNP (N), NPQ (O), ${\text{F}}_{\text{m}}^{\prime }$/${\text{F}}_{\text{v}}^{\prime }$ (P), Φ_PSII_ (Q), and ETR (R) in dark-adapted *C. grandis* and *C. sinensis* leaves**. Bars represent means ± SE (*n* = 7–15). Differences among the 10 treatments were analyzed by two (species) × five (pH) factorial ANOVA. Different letters above the bars indicate a significant difference at *P* < 0.05.

As shown in Figure 7, the F_o_, F_m_, M_o_, ABC/RC, DI_o_/RC, DI_o_/ABS, qNP, and NPQ all increased, and whereas the F_v_/F_m_, ET_o_/ABS, RE_o_/ABS, P_2G_, PI_tot, abs_, qP, F_m_′/F_v_′, Φ_PSII_, and ETR all decreased as the pH increased from 2.5 to 3, with further increasing pH there was hardly any change in all these parameters. Nonetheless, the F_v_ did not greatly change in response to pH. All these parameters were similar between the two citrus species at pH 3, 4, 5, or 6, but the pH 2.5-induced changes in F_o_, F_v_, F_m_, M_o_, ABC/RC, DI_o_/RC, DI_o_/RC, F_v_/F_m_, RE_o_/ABS, P_2G_, PI_tot, abs_, and ETR were slightly greater in *C. grandis* than in *C. sinensis* leaves.

**Figure 8:**
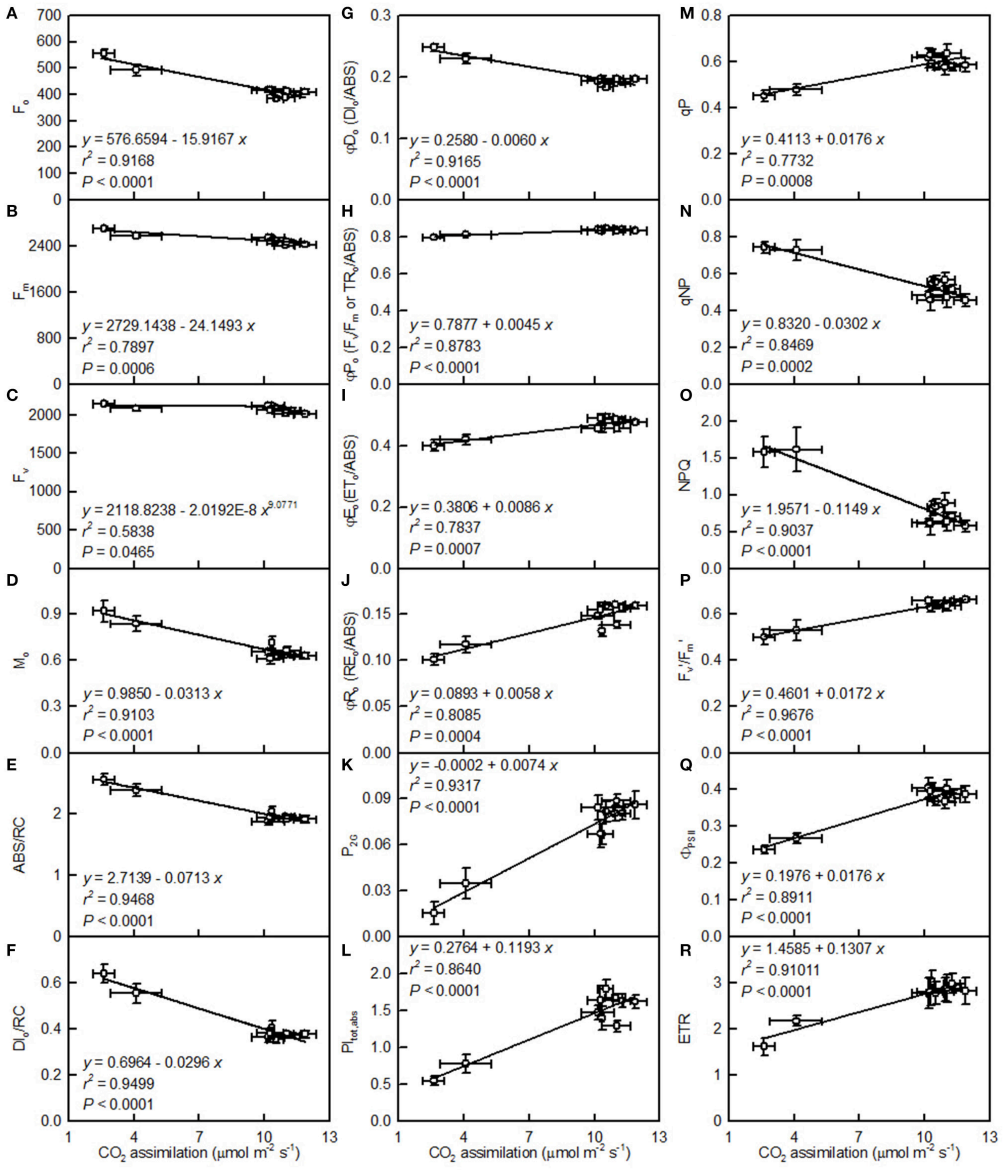
**Leaf CO_2_ assimilation in relation to F_o_ (A), F_m_ (B), F_v_ (C), M_o_ (D), ABS/RC (E), DI_o_/RC (F), DI_o_/ABS (G), F_v_/F_m_ (H), ET_o_/ABS (I), RE_o_/ABS (J), P_2G_ (K), PI_tot, abs_ (L), qP (M), qNP (N), NPQ (O), ${\text{F}}_{\text{m}}^{\prime }$/${\text{F}}_{\text{v}}^{\prime }$ (P), Φ_PSII_ (Q), and ETR (R)**. Points represent means ± SE for the independent variable (*n* = 4) and the dependent variables (*n* = 7–15). Data for CO_2_ assimilation are from Figure 3. Data for the 18 fluorescence parameters are from Figure 7. Data for the two citrus species were pooled together.

Leaf CO_2_ assimilation decreased with increasing F_o_, F_m_, F_v_, M_o_, ABC/RC, DI_o_/RC, DI_o_/ABS, qNP, or NPQ, whereas it increased with increasing F_v_/F_m_, ET_o_/ABS, RE_o_/ABS, P_2*G*_, PI_tot, abs_, qP, F_m_′/F_v_′, Φ_PSII_, or ETR (Figure 8).

### Effects of pH on RWC, H_2_O_2_ production, electron leakage, total soluble proteins in roots and leaves and specific leaf weight

**Figure 9:**
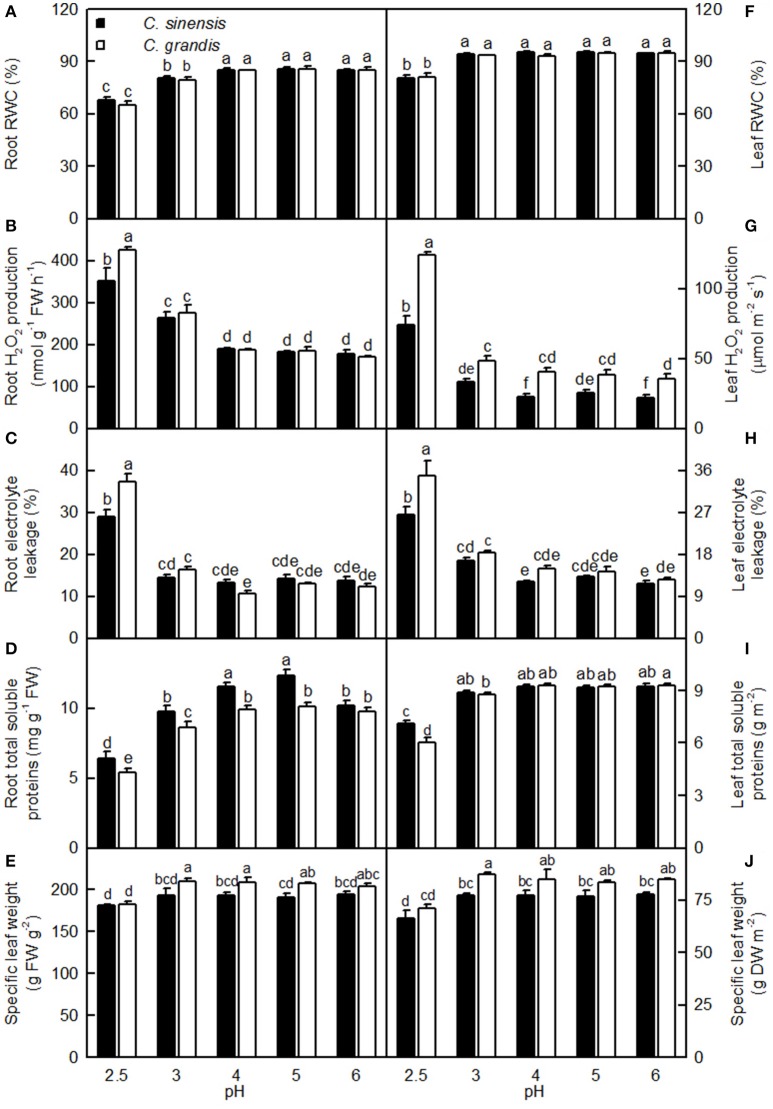
**Effects of pH on root (A–D) and leaf (F–I) relative water content (RWC, A,F), H_2_O_2_ production (B,G), electrolyte leakage (C,H), concentrations of total soluble proteins (D,I), and specific leaf weight expressed on a fresh weight (FW, E) or dry weight (DW, J) basis in the *C. sinensis* and *C. grandis* seedlings**. Bars represent means ± SE (*n* = 4). Differences among the 10 treatments were analyzed by two (species) × five (pH) factorial ANOVA. Different letters above the bars indicate a significant difference at *P* < 0.05.

Both pH 2.5 and 3 decreased the root RWC, while only pH 2.5 lowered the leaf RWC. Root and leaf RWCs were similar between the two citrus species at each given pH (Figures 9A,F).

Both pH 2.5 and 3 increased the root H_2_O_2_ production, while only pH 2.5 enhanced the leaf H_2_O_2_ production. Root (Leaf) H_2_O_2_ production was significantly higher in *C. grandis* than in *C. sinensis* at pH 2.5 (2.5, 3, 4, and 6), but similar between the citrus species at pH 3–6 (pH 5; Figures 9B,G).

Root and leaf electrolyte leakage increased as the pH increased from 2.5 to 3, after which leakage remained relatively stable under increasing pH. Root and leaf electrolyte leakage was higher in *C. grandis* than in *C. sinensis* at pH 2.5, but it was similar between the citrus species at pH 3–6 (Figures 9C,H).

For *C. grandis*, the total soluble protein level in roots increased as the pH increased from 2.5 to 4, after which it remained unchanged with increasing pH. For *C. sinensis*, the total soluble protein level in roots was lowest at pH 2.5, intermediate at pH 3 and 6, and highest at pH 4 and 5. The total soluble protein level in leaves of the two citrus species increased as the pH increased from 2.5 to 3, but these levels were little changed with increasing pH. The total soluble protein levels in roots and leaves were higher in *C. grandis* than in *C. sinensis*, or they were statistically similar between the two species at each given pH (Figures 9D,I).

The specific leaf weight was decreased at pH 2.5 and it was higher in *C. grandis* than in *C. sinensis*, or it was similar between the two species at each given pH irrespective of how the data were expressed (Figures 9E,J).

**Figure 10:**
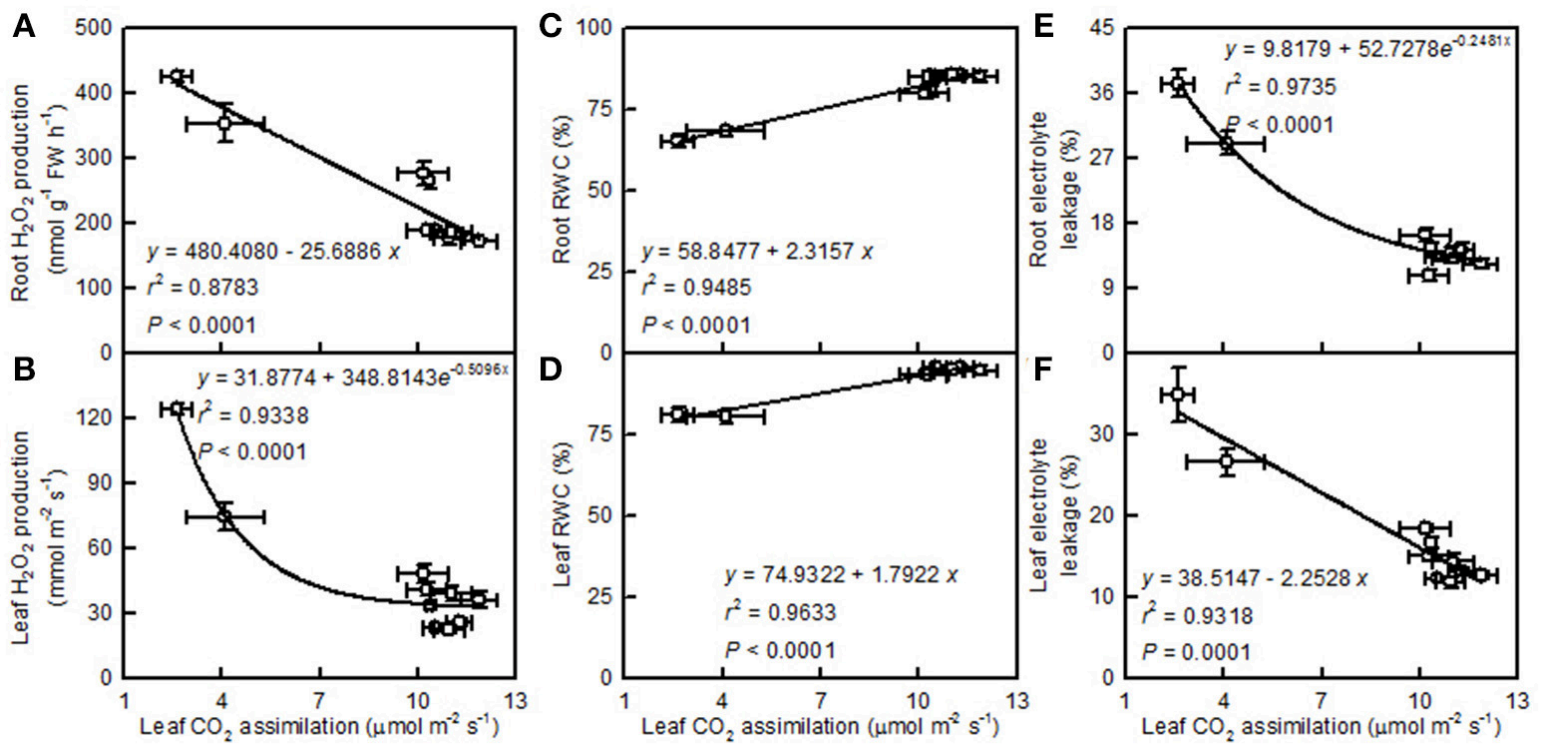
**Leaf CO_2_ assimilation in relation to root (A,C,E) and leaf (B,D,F) H_2_O_2_ production (A,B), RWC (C,D), and electrolyte leakage (E,F)**. Points represent means ± SE for the independent variable (*n* = 4) and the dependent variables (*n* = 4). Data for CO_2_ assimilation came from Figure 3. Data for H_2_O_2_ production, RWC, and electrolyte leakage came from Figure 9. Data for the two citrus species were pooled together.

Leaf CO_2_ assimilation decreased with increasing root and leaf H_2_O_2_ production or electrolyte leakage, but it increased with increasing root and leaf RWC (Figure 10).

### Effects of pH on element concentrations, uptake, and distributions

**Figure 11:**
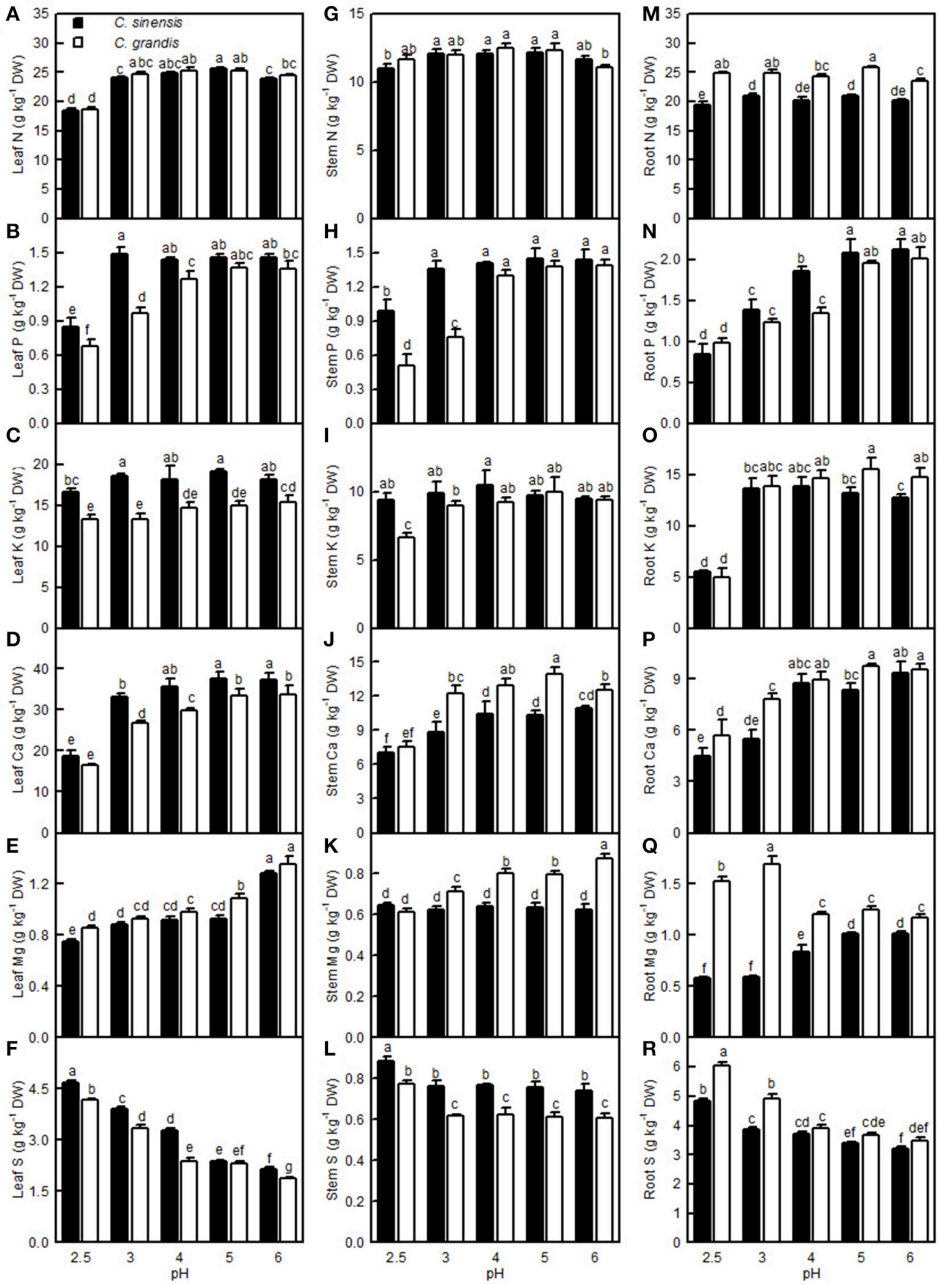
**Effects of pH on the N (A,G,M), P (B,H,N), K (C,I,O), Ca (D,J,P), Mg (E,K,Q), and S (F,L,R) concentrations in *C. sinensis* and *C. grandis* leaves, stems, and roots**. Bars represent means ± SE (*n* = 4). Differences among the 10 treatments were analyzed by two (species) × five (pH) factorial ANOVA. Different letters above the bars indicate a significant difference at *P* < 0.05.

The leaf N level was lower at pH 2.5 than at pH 3–6, but the stem and root N levels remained little changed over the range of pH 2.5–6. The P level in *C. grandis* (*C. sinensis*) leaves and stems increased as the pH increased from 2.5 to 4 (3), but it went unchanged with increasing pH. The root P level increased as the pH increased from 2.5 to 5, but it then kept stable with increasing pH. The K concentration in the *C. sinensis* leaves and stems and in the *C. grandis* leaves displayed little change in the range of pH 2.5–6; however, the K level in the *C. sinensis* roots and in the *C. grandis* stems and leaves was lower at pH 2.5 than at pH 3–6. Generally viewed, the Ca levels in the leaves, stems, and roots all increased as the pH increased from 2.5 to 4, after which they were relatively stable with increasing pH. The Mg level in the *C. grandis* leaves and stems and in the *C. sinensis* leaves decreased with decreasing pH, but the Mg level in the *C. sinensis* stems did not change in response to pH. The Mg level in the *C. sinensis* roots was reduced at pH 2.5, 3, and 4, but especially at pH 2.5 and 3, while its level in the *C. grandis* roots was elevated at pH 2.5 and pH 3, though especially at pH 3. Leaf and root S decreased with increasing pH, while the stem S level was higher at pH 2.5 than at the other pH treatments. Leaf P, K, Ca, and S, stem P, K, and S, and root P levels were all higher in *C. sinensis* than in *C. grandis* seedlings; or similar between the two citrus species at each given pH. Conversely, the leaf Mg, stem Ca and Mg, and root N, K, Ca, Mg, and S levels were all lower in *C. sinensis* than in *C. grandis* seedlings, or they were similar between the two citrus species at each given pH (Figure 11).

**Figure 12:**
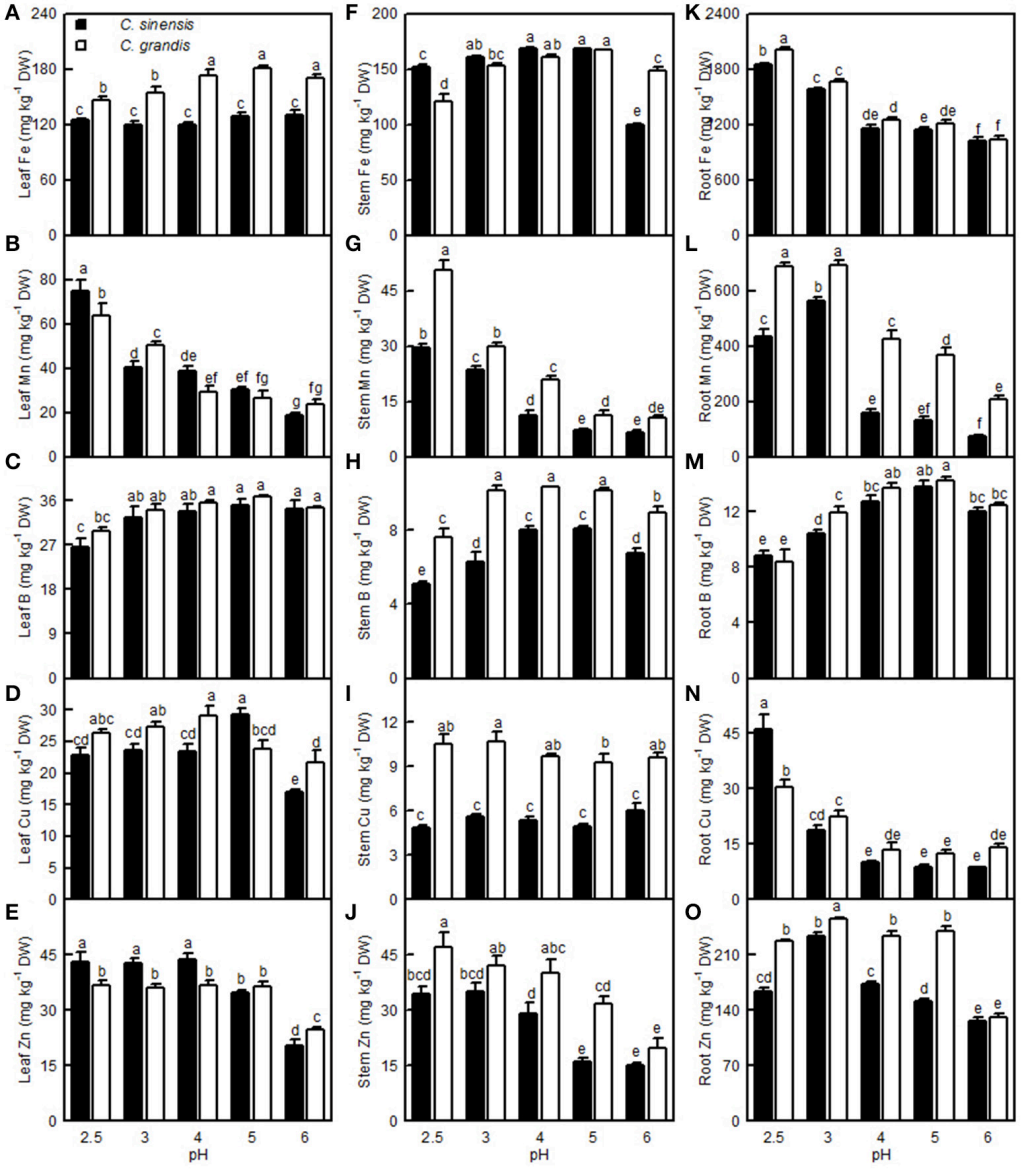
**Effects of pH on the Fe (A,F,K), Mn (B,G,L), B (C,H,M), Cu (D,I,N), and Zn (E,J,O) concentrations in the *C. sinensis* and *C. grandis* leaves, stems, and roots. Bars represent means ± SE (*n* = 4)**. Differences among the 10 treatments were analyzed by two (species) × five (pH) factorial ANOVA. Different letters above the bars indicate a significant difference at *P* < 0.05.

The Fe level in the *C. grandis* leaves was lower at pH 2.5 and 3 than at pH 4–6, while the Fe level in the *C. sinensis* leaves did not differ among the five pH treatments. The Fe level in the *C. sinensis* (*C. grandis*) stems increased as the pH increased from 2.5 to 3 (4), but it then kept relatively stable with increasing pH, though it decreased at pH 6. The root Fe concentration decreased with increasing pH. Leaf and stem Mn levels decreased with increasing pH. The root Mn level increased as the pH decreased from 6 to 3, then it decreased or went unchanged at pH 2.5. Leaf B concentration in the two citrus species was decreased only at pH 2.5. The B level in the *C. sinensis* (*C. grandis*) stems increased as the pH increased from 2.5 to 4 (3), but then it went unchanged with increasing pH, though it decreased at pH 6. Although the root B concentration increased as the pH increased from 2.5 to 5, it decreased at pH 6. The Cu level in the *C. grandis* leaves increased as the pH decreased from 6 to 4, after which it was little changed with decreasing pH; the Cu level in the *C. sinensis* leaves was highest at pH 5 and lowest at pH 6. Root Cu level in the two citrus species decreased as the pH increased from 2.5 to 4, but it then remained stable with increasing pH. The Zn level in the *C. sinensis* leaves and stems were lower at pH 5 and 6 than at pH 2.5, 3, and 4, while its level in the *C. grandis* leaves and stems were lower at pH 6 than at pH 2.5–5. The Zn level in the *C. sinensis* roots increased as the pH decreased from 6 to 3, but it then decreased at pH 2.5; the Zn level in *C. grandis* roots was highest at pH 3 and lowest at pH 6. Generally viewed, the leaf Fe, Mn, B and Cu, stem Fe, Mn, B, Cu and Zn, root Fe, B, Mn, and Zn concentrations all were higher in *C. grandis* than in *C. sinensis*, or they were similar between the two citrus species at each given pH. The exceptions to this generalization were that the Mn (Cu) level was higher in *C. sinensis* than in *C. grandis* leaves at pH 2.5 (5), and the Fe level was higher in *C. sinensis* than in *C. grandis* stems at pH 2.5. By contrast, the leaf Zn and root Cu concentrations were higher in the *C. sinensis* than in those of *C. grandis*, or they were similar between the two citrus species at pH 2.5–5, albeit leaf Zn lower was lower in the *C. sinensis* vs. *C. grandis* at pH 6 (Figure 12).

**Figure 13:**
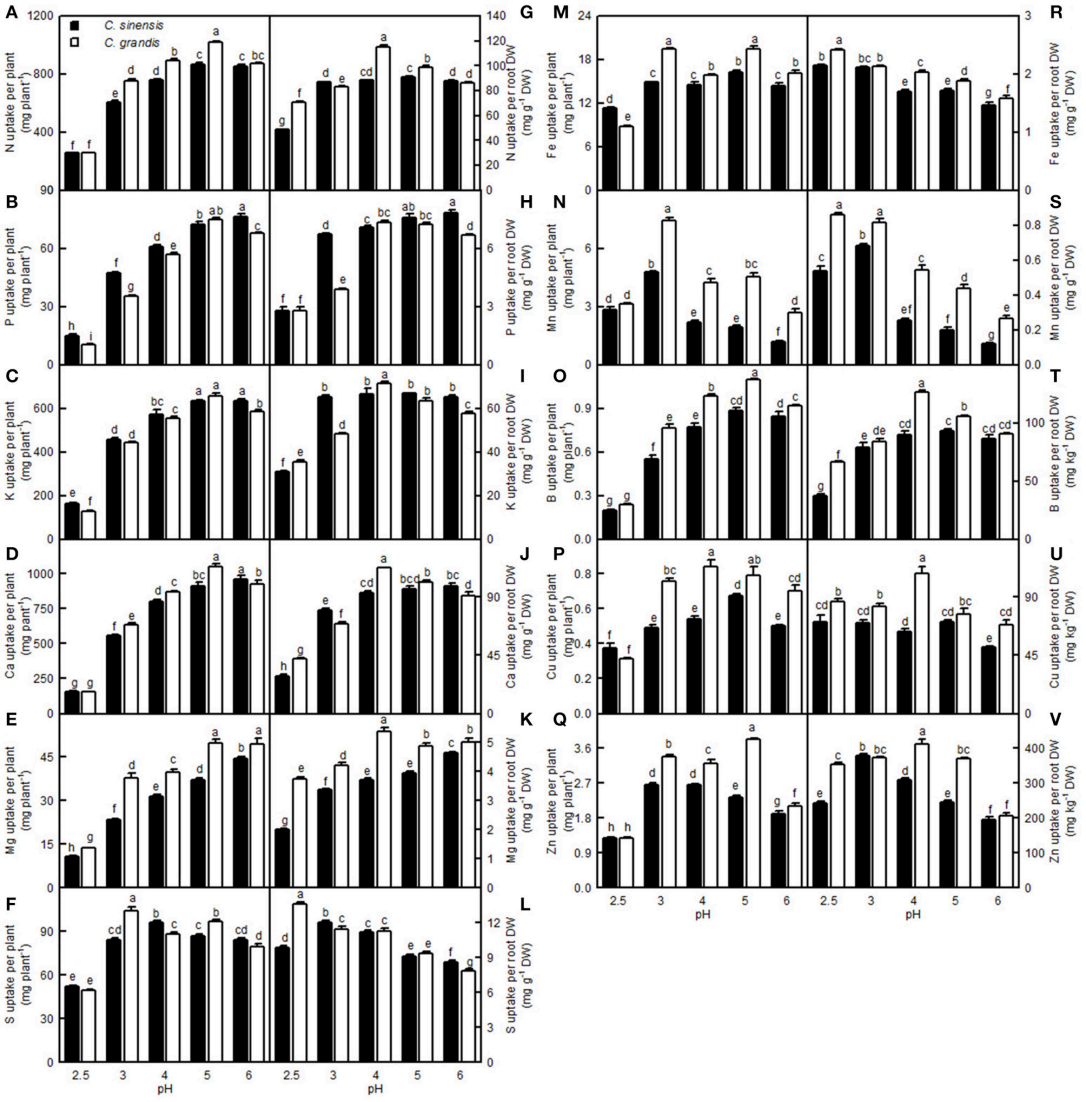
**Effects of pH on mineral element uptake per plant (A–F,M–Q) and per root DW (G–L,R–V)**. Bars represent means ± SE (*n* = 4). Differences among the 10 treatments were analyzed by two (species) × five (pH) factorial ANOVA. Different letters above the bars indicate a significant difference at *P* < 0.05.

For *C. sinensis*, the N, P, K, Ca, Mg, and B uptake per plant increased as the pH increased from 2.5 to 5, then continued to rise or kept unchanged with increasing pH; For *C. grandis*, these elemental uptake per plant increased as the pH increased from 2.5 to 5, but then it went unchanged or decreased with increasing pH. The Mn uptake per plant in the two citrus species increased as the pH increased from 2.5 to 3, but it then decreased with increasing pH. Treatment with pH 2.5 decreased the S, Fe, Cu, and Zn uptake per plant compared with the corresponding uptake at pH 5 (Figures 13A–F,M–Q).

Compared with pH 5, treatment with pH 2.5 decreased the N, P, K, Ca, Mg, and B uptake per root DW, whereas it increased the S, Fe, Mn, and Zn uptake per root DW; however, pH 2.5 did not influence Cu and Zn uptake per root DW (Figures 13G–L,R–V).

**Figure 14:**
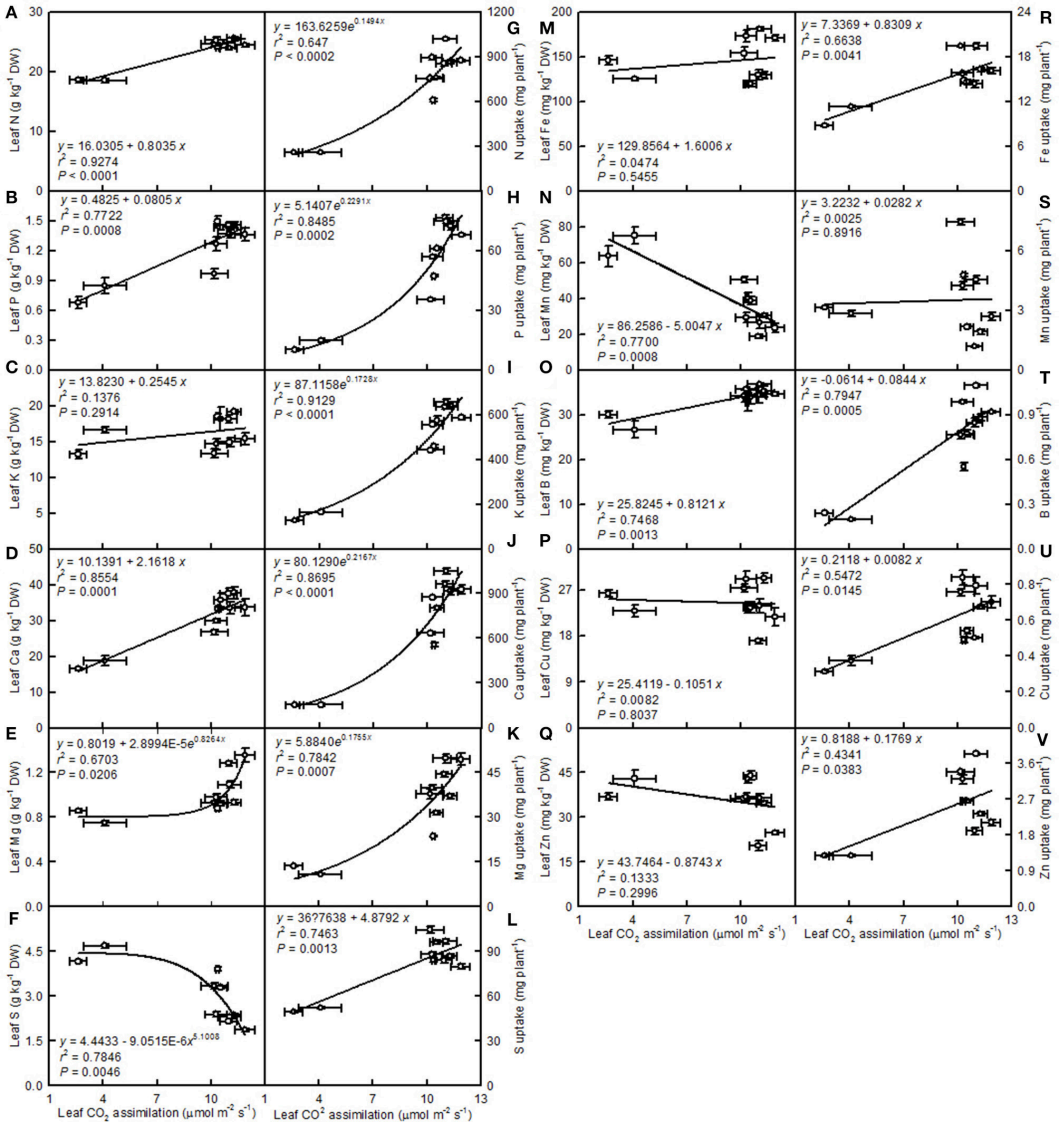
**Leaf CO_2_ assimilation in relation to the mineral element concentrations in leaves (A–F,M–Q) and their uptake per plant (G–L,R–V)**. Points represent means ± SE for the independent variable (*n* = 4) and the dependent variables (*n* = 4). Data for CO_2_ assimilation came from Figure 3. Data for the mineral element concentrations (mineral element uptake per plant) came from Figures 11–13). Data for the two citrus species were pooled together.

Leaf CO_2_ assimilation increased with increasing leaf N, P, Ca, Mg, Fe, or B, whereas it decreased with increasing leaf S, Mn, Cu, or Zn—it did not display a significant relationship with leaf K. Except for the Mn uptake per plant, the leaf CO_2_ assimilation increased with increasing uptake per plant of the other elements (Figure 14).

Compared with pH 5, treatment with pH 2.5 lowered all the element distributions in the *C. sinensis* leaves and the S, Fe, and Cu distributions in the *C. sinensis* stems; it increased, or did not affect, the 11 element distributions in the *C. sinensis* roots and the N, P, K, Mg, Mn, B, and Zn distributions in the *C. sinensis* stems. Compared with pH 5, pH 2.5 decreased or did not influence the K distribution in the stems and roots and the distributions of the other 10 elements in the leaves and stems; pH 2.5 increased or did not influence the K distribution in the leaves and the distributions of the other 10 elements in the roots of the *C. grandis* seedlings (Figures S1, S2).

## Discussion

Low pH very often affects the uptake of nutrients and water by plants (Kamaluddin and Zwiazek, 2004; Bian et al., 2013). As expected, pH 2.5 lowered the water uptake in citrus, as indicated by the reduced root and leaf RWC (Figures 9A,F). This result is supported by the finding that the water content was decreased in low pH-treated *Eucalyptus* roots, stems, and leaves (Yang M. et al., 2011). As shown in Figures 9–13, the uptakes of mineral nutrients were greatly altered at pH 2.5. Compared with pH 5, the pH 2.5 lowered N, P, K, Ca, and Mg uptake per plant or root DW, and the S uptake per plant. Low pH (4.0 relative to 7.0) induced decreases in the N, P, K, Ca, and Mg uptake per plant in *V. faba* (Schubert et al., 1990). Similarly, Malkanthi et al. (1995) observed that a pH 3.8 (relative to 5.5) decreased the K, Ca, and Mg uptake per plant in wheat, barley, and chili, and likewise in cowpea for Ca and Mg uptake per plant. However, the uptake of N, P, K, Ca, and Mg in sago palm seedlings was not changed in the range of pH 3.6–5.7 over a 4.5-month period (Anugoolprasert et al., 2012). Thus, it appears that the effects of low pH on macronutrient uptake per plant depend on both the plant species identity and the H^+^ strength (i.e., pH value).

On the whole, apart from a few exceptions, the concentrations of N, P, K, Ca, and Mg were decreased in the pH 2.5-treated *C. grandis* and *C. sinensis* roots, stems, and leaves (Figure 11). This agrees with the report that pH 3 decreased Ca and Mg levels in *Picea abies* roots and needles (George et al., 2012); that P, K, and Mg levels were lowered in the low pH-treated *V. faba* roots and shoots (Schubert et al., 1990); that the levels of K, Ca, and Mg in the roots and tops of wheat, barley, and chili were lower at pH 3.8 than at pH 5.7 (Malkanthi et al., 1995); and that P and Ca levels in pummelo leaves increased linearly with increasing soil pH (Li et al., 2015). However, the levels of N, P, K, Ca, and Mg in the roots, leaflets, petioles, and whole plant of sago palm seedlings did not differ among pH 3.6, 4.5, and 5.7 (Anugoolprasert et al., 2012). The concentrations of N, P, K, Ca, and Mg might have been reduced in sago palm seedlings if the pH was lower than pH 3.6, because the concentration of N, P, K, Ca, and Mg in citrus roots, stems, and leaves were greatly reduced at pH 2.5 but little affected at pH 4 relative to pH 5 (Figure 11). In contrast, the S level was increased in the low pH-treated *C. grandis* and *C. sinensis* roots, stems, and leaves (Figures 11F,L,R), which is consistent with the finding that the S concentration in the tops of ginger, maize, wheat, French bean, and tomato plants was higher at pH 3.3 than at pH 4.0 (Islam et al., 1980).

So far, however, there is little published information available on the effects of low pH on plant micronutrients. H^+^-toxicity is thought to inhibit the uptake of cations (George et al., 2012). However, treatment with pH 2.5 did not lower Fe, Cu, Mn, and Zn uptake per root DW in the two citrus species, or the Mn uptake per plant in *C. sinensis*, when compared with pH 5 (Figures 13N,R,S,U,V). This result may be related to the insensitivity of citrus plants to acidic soils, as reported previously by Yuda and Okamoto (1965). Interestingly, the B uptake per plant or per root DW was reduced by a low pH (Figures 13O,T). This result is supported by a work showing that B could alleviate low pH-induced damage in *Arabidopsis* roots (Koyama et al., 2001).

The Fe, Mn, Cu, and Zn concentrations in the *C. grandis* and *C. sinensis* roots, stems, and leaves were all higher at pH 2.5 than at pH 5, or they were similar between the two treatments, though there was a lower level of Fe detected in the *C. grandis* leaves at pH 2.5 than at pH 5 (Figures 12A,B,D–G,I–L,N–Q). The observed higher Fe, Mn, Cu, and Zn concentrations in the pH 2.5-treated *C. grandis* and *C. sinensis* roots, stems, and leaves might be associated with a reduced dilution due to decreased growth (Figure 1) and with higher uptake per root DW (Figures 13R,S,U,V). As shown in Figures 12A,D,F,I,K,N, the root Fe and Cu concentrations were higher at pH 2.5 than those at the other treatment levels of pH, while no such results were observed for the leaf and stem Fe and Cu concentrations; this may be explained by the increased Fe and Cu distributions in the roots, and the decreased or unchanged Fe and Cu distributions in the leaves and stems, at pH 2.5 (Figures S2A,D,F,I,K,N). By contrast, the B level was decreased in the pH 2.5-treated *C. grandis* and *C. sinensis* roots, stems, and leaves (Figures 12C,H,M) likely due to the decreased B uptake per plant or root DW (Figures 13O,T).

In this experiment, many of the fibrous roots became rotten and the living roots turned abnormally dark brown when exposed to pH 2.5 (Figures 2A,D). Thus, it is reasonable to presume that H^+^-toxicity may directly damage citrus roots, thus affecting the uptake of vital mineral nutrients and water.

Our results showed that pH 2.5 lowered the root, stem, leaf, and whole plant DW (Figures 1, 2). The low pH-induced poor growth of citrus seedlings may be due to the combined interplay of direct H^+^-toxicity—as shown by the damaged roots (Figures 2A,D)—deficiencies of macronutrients—as indicated by the decreased N, P, K, Ca, and Mg concentrations (Figures 11A–E)—and uptake per plant or root DW (Figures 13A–E,G–K), and the decreased water uptake—as indicated by the decreased root and leaf RWC (Figures 9A,F).

In spite of the reduced growth at pH 2.5, no seedling deaths occurred in the two citrus species at each given pH during the entire experiment. Similar results have been obtained for several citrus rootstocks (Fang, 2011; Fang et al., 2011), as well as for *C. sinensis* seedlings (Guest and Chapman, 1944). Based on the present results, we conclude that the two citrus species studied were insensitive to low pH. This above conclusion is supported by the fact that most of physiological parameters monitored in Figures 3, 4, 7, 9 were altered only at pH 2.5, and that pH 4 had almost no influence on these parameters and the OJIP transients (Figure 6).

As shown in Figure 2B, mottled bleached leaves were observed only in the pH 2.5-treated *C. grandis* seedlings (Figure 2B). Furthermore, the pH 2.5-induced alterations of many physiological parameters shown in Figures 3, 4, 7, 9, and of the JIP transients (Figure 6), were slightly greater in *C. grandis* than in *C. sinensis* leaves. Evidently, when the results are taken together, seedlings of *C. sinensis* had a slightly higher tolerance to a low pH than did those of *C. grandis*. However, the difference in low pH tolerance between the *C. grandis* and *C. sinensis* species is apparently lower than the difference between them in their Al-tolerance (Yang L. T. et al., 2011; Jiang et al., 2015; Li et al., 2016). This latter discrepancy is supported by a study showing that plant races were separately adapted to Al^3+^ or low pH- (H^+^-) toxicity (Kidd and Proctor, 2001).

We found that pH 2.5 greatly inhibited the CO_2_ assimilation in *C. grandis* and *C. sinensis* leaves, and that this inhibition was slightly greater in *C. grandis* than in *C. sinensis* leaves (Figure 3A). The pH 2.5-induced decrease in leaf CO_2_ assimilation could not be explained only by decreased stomatal conductance, because the intercellular CO_2_ concentration increased and stayed unchanged in pH 2.5-treated *C. granddis* and *C. sinensis* leaves, respectively (Figure 3C), and because leaf CO_2_ assimilation decreased with the increasing intercellular CO_2_ concentration (Figure 5B). Thus, the pH 2.5-induced decrease in leaf CO_2_ assimilation in citrus may be primarily driven by non-stomatal factors.

In this context, the pH 2.5-induced decreases in Chl a, Chl b, and Chl a+b were probably not the main factor inhibiting leaf CO_2_ assimilation because their reductions were much lower than that for leaf CO_2_ assimilation (Figures 3A, 4A–C). This conclusion is supported by our results showing that DI_o_/RC, DI_o_/ABS, NPQ, and qNP were all elevated in the pH 2.5-treated *C. grandis* and *C. sinensis* leaves (Figures 7F,G,N,O).

The observed positive ΔL-band at ca. 130 μs in the OJIP transients from the pH 2.5-treated leaves (Figures 6E,J) suggested that the grouping (stability) of the PSII units and the energy exchange between the independent PSII units were both reduced (Strasser et al., 2004; Liao et al., 2015). This interpretation is further supported by our result that P_2G_ was decreased in the pH 2.5-treated leaves (Figure 7K). The appearance of a positive ΔK-band at 300 μs in the OJIP transients from the pH 2.5-treated leaves (Figures 6C,H) indicated that the oxygen evolving complex (OEC) had been damaged (Srivastava et al., 1997). The observed positive ΔJ- and ΔI-bands at 2 ms and 30 ms, respectively, in the OJIP transients from the pH 2.5-treated leaves (Figures 6C,H) suggested that the reduction of the PSII acceptor side had been elevated (Strasser et al., 2004). The amount of electrons from the RCs at the acceptor side depends on both the capacity of electron donation to the RCs and the capacity of the electron transport chain from the RCs to the electron acceptors. Based on these results, we conclude that at pH 2.5, the PSII acceptor side was more severely damaged than was the PSII donor side. We observed that pH 2.5 led to increased DI_o_/RC, decreased F_v_/F_m_ and ET_o_/ABS (Figures 7F,H,I), and altered the OJIP transients (Figure 6) in leaves, together indicating that photoinhibition damaged the PSII complexes in these citrus leaves (Maxwell and Johnson, 2000; Force et al., 2003). In the present study, the pH 2.5-induced decrease in F_v_/F_m_ was caused by an increased F_o_, since the F_m_ slightly increased with decreasing pH (Figures 7A,B). The observed higher F_o_ in the pH 2.5-treated leaves was likely associated with an increased inactivation of the PSII RCs, as increased by the decreased qP (Figure 7M), and with the enhanced damage to OEC, as indicated by the positive ΔK-band (Figures 6C,H). Furthermore, the higher F_o_ may have arisen from the pH 2.5-induced accumulation of reduced Q_A_ (Bukhov et al., 1990), as indicated by the increased M_o_ (Figure 7D). In addition, the pH 2.5-treated leaves displayed decreased RE_o_/ABS, PI_tot, abs_, F_m_′/F_v_′, Φ_PSII_, and ETR (Figures 7J,L,P–R). Obviously, treatment with pH 2.5 impaired the whole electron transport chain from the donor side of PSII to the reduction of the PSII end acceptors, thus decreasing ETR. Regression analysis showed that leaf CO_2_ assimilation increased with increasing F_v_/F_m_, ET_o_/ABS, RE_o_/ABS, P_2G_, PI_tot, abs_, qP, F_m_′/F_v_′, Φ_PSII_, or ETR, (Figures 8H–M,P–R). Based on these results, we conclude that pH 2.5 damaged the whole photosynthetic electron transport chain, thus inhibiting leaf CO_2_ assimilation in seedlings of these two citrus species.

Light-driven ROS production can cause oxidative damage to vital photosynthetic components and thereby inhibit photosynthesis (Foyer and Shigeoka, 2011). We observed that pH 2.5 greatly increased the H_2_O_2_ production and the electrolyte leakage in *C. grandis* and *C. sinensis* leaves, though more so in the *C. grandis* leaves (Figures 9G,H), and that leaf CO_2_ assimilation decreased with increasing leaf H_2_O_2_ production and electrolyte leakage (Figures 10B,F). Hence, the observed higher ROS production may be responsible for the pH 2.5-induced inhibition of photosynthesis in citrus leaves.

The leaf photosynthetic rate decreases with decreasing leaf RWC (Lawlor, 2002). However, the relative importance of stomatal and non-stomatal limitations to photosynthesis under water stress is not yet fully understood. Typically, as the RWC decreases, the stomatal limitation of photosynthesis will also decrease and the metabolic limitation will increase (Lawlor, 2002; Zhou et al., 2007), which entails limitations to ribulose-1,5-disphosphate (RuBP) regeneration (Gunasekera and Berkowitz, 1993), photophosphorylation (Tezara et al., 1999), and Rubisco activity (Maroco et al., 2002; Parry et al., 2002). Zhou et al. (2007) observed that water stress decreased photosynthetic rate, Rubisco activity, the energy flux via linear electron transport, and increased ΔpH- and xanthophyll-mediated thermal dissipation. Our results showed that the pH 2.5-induced decrease in leaf CO_2_ assimilation (Figure 3A) was accompanied by decreases in root and leaf RWC (Figures 9A,F), leaf Rubisco activity (Figure 3F) and ETR, and by an increase in NPQ (Figures 7O,R). Furthermore, leaf CO_2_ assimilation decreased with decreasing root RWC, leaf RWC (Figures 10C,D), Rubisco activity (Figure 5C), or ETR (Figure 8R), and with increasing NPQ (Figure 8O); leaf Rubisco activity (*y*) increased with increasing leaf RWC (*y* = −61.1653 + 0.8351*x, r*^2^ = 0.9174, *P* < 0.0001). Thus, it is reasonable to assume that a low pH lowered the water uptake and induced water stress, thus inhibiting photosynthesis in the *C. grandis* and *C. sinensis* leaves.

A study has shown that the base cation-induced increase in sugar maple photosynthesis on acid soils was associated with an improved foliar nutrient status (St Clair and Lynch, 2005). Ellsworth and Liu (1994) suggested that leaf photosynthesis of sugar maple on acidic soils was co-limited by N and Ca, or by interactions of Ca with other nutrients, such as Mg. We observed that leaf CO_2_ assimilation decreased with increasing leaf N, P, Ca, or Mg concentrations (N, P, Ca, or Mg uptake per plant) (Figures 14A,B,D,E,G,H,J,K). Thus, the pH 2.5-induced decreases in these nutrients might be responsible for the observed lower leaf CO_2_ assimilation.

Our results also showed that the growth of seedlings (Figures 1, 2) and the status of many of their physiological parameters (Figures 3, 4, 7, 9, 11, 12) reached their maximum at pH 5. This seems to contradict the early view that serious problems for citrus might arise when the soil pH was 5.0 or lower (Chapman, 1968). In our study, citrus seedlings were grown under favorable conditions of mineral nutrients and the direct toxicity of H^+^ might be the primary cause for the poor seedling growth. However, a significant difference might also occur when citrus are grown on acidic soils due to the increased solubility of Al and Mn, and/or decreased availability of P, Ca, Mg, and Mo (George et al., 2012; Kochian et al., 2015; Li et al., 2015). Thus, the optimum pH for citrus might be higher in a soil culture than when grown in solution or a sand culture (Yuda and Okamoto, 1965). These findings indicate that suitable fertilizers might alleviate the toxicity of acidic soils upon citrus. Adjusting the soil nutrients via careful fertilization should contribute to greater harvest yield and the sustainable management of citrus across a range of acidic soils.

## Conclusion

Our results demonstrate that citrus seedlings were insensitive to low pH, and that *C. sinensis* is slightly more tolerant to this low pH than is *C. grandis*. H^+^-toxicity could directly damage the citrus roots, thus affecting their uptake of mineral nutrients and water. The results suggest that the low pH-induced inhibition of growth was caused by the combination of H^+^-toxicity, deficiencies of nutrients, and decreased water uptake. Here, only pH 2.5 noticeably inhibited leaf CO_2_ assimilation, which was probably due to the combination of an impaired photosynthetic electron transport chain, increased ROS production, and decreased uptake of water and nutrients. In sum, these findings increase our understanding of the factors by which a low pH can decrease citrus growth, and of the mechanisms by which low pH inhibits leaf CO_2_ assimilation.

## Author contributions

AL performed most of the experiment and drafted the manuscript; JZ participated in the measurements of photosynthesis and fluorescence; LY participated in the direction of this study; XY and NL participated in the analysis of the nutrient elements; LT and DL participated in the cultivation of the experimental seedlings; LC designed and directed the study and also revised the manuscript. All authors have read and approved the final manuscript.

## Funding

This work was financially supported by an earmarked fund for the China Agriculture Research System (No. CARS27).

### Conflict of interest statement

The authors declare that the research was conducted in the absence of any commercial or financial relationships that could be construed as a potential conflict of interest.
