# Supplementary material for: Effects of Low pH on Photosynthesis, Related Physiological Parameters, and Nutrient Profiles of Citrus
Source: Front Plant Sci. 2017 Feb 21;8:185. doi: 10.3389/fpls.2017.00185 (PMC5318377; doi:10.3389/fpls.2017.00185)
Supplement: Supplementary file 1 [file DataSheet1.PDF]

SUPPLEMENTARY MATERIAL

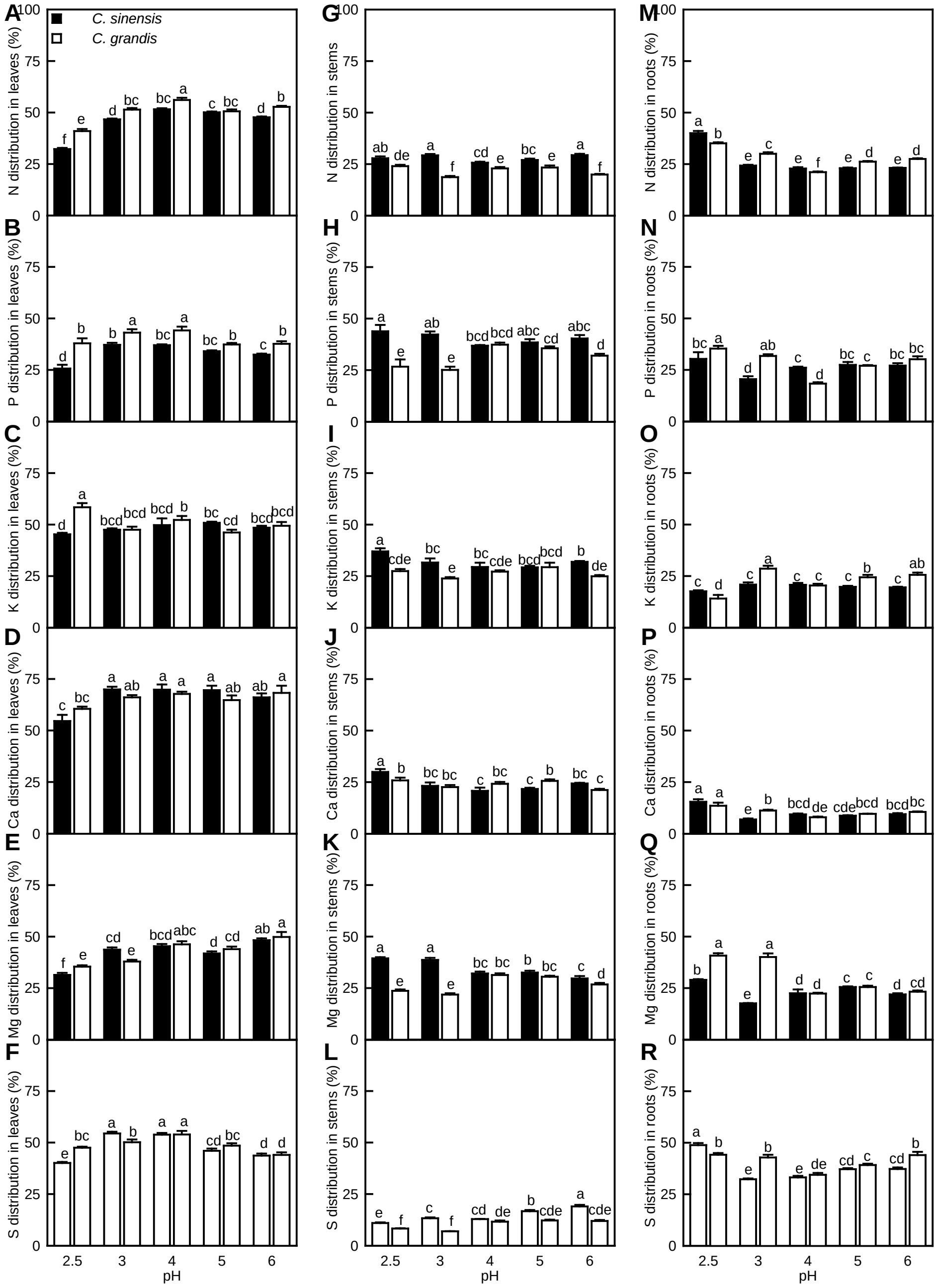

FIGURE S1 | Effects of pH on N, P, K, Ca, Mg and S distributions in *C. sinensis* and *C. grandis* leaves (A-F), stems (G-L) and roots (M-R). Bars represent means ± SE (n = 4). Differences among 10 treatments were analyzed by two (species) × five (pH) ANOVA. Different letters above the bars indicate a significant difference at $P < 0.05$.

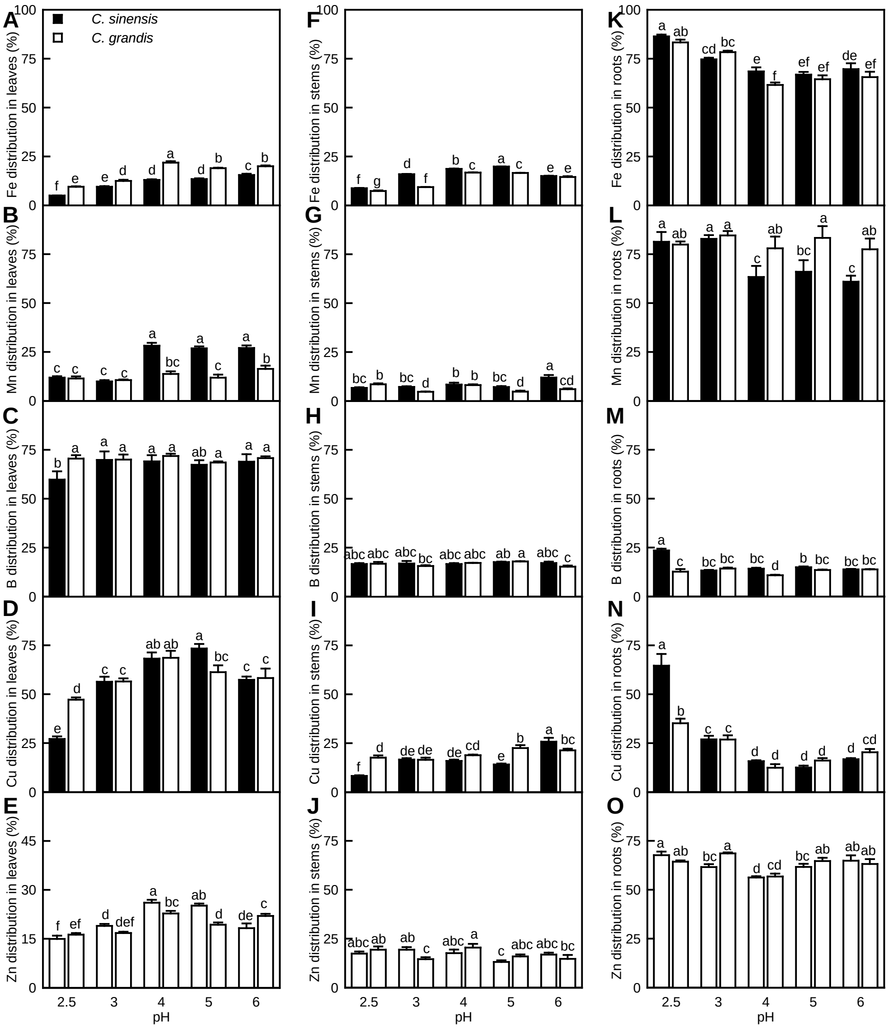

FIGURE S2 | Effects of pH on Fe, Mn, B, Cu and Zn distributions in *C. sinensis* and *C. grandis* leaves (A-E), stems (F-J) and roots (K-O). Bars represent means ± SE (n = 4). Differences among 10 treatments were analyzed by two (species) × five (pH) ANOVA. Different letters above the bars indicate a significant difference at P < 0.05.
